# Causal Interrogation of Neuronal Networks and Behavior through Virally Transduced Ivermectin Receptors

**DOI:** 10.3389/fnmol.2016.00075

**Published:** 2016-08-30

**Authors:** Horst A. Obenhaus, Andrei Rozov, Ilaria Bertocchi, Wannan Tang, Joachim Kirsch, Heinrich Betz, Rolf Sprengel

**Affiliations:** ^1^Sprengel Research Group, Department of Molecular Neurobiology, Max Planck Institute for Medical ResearchHeidelberg, Germany; ^2^OpenLab of Neurobiology, Kazan Federal UniversityKazan, Russia; ^3^Division of Neuro- and Sensory Physiology, Institute of Physiology and Pathophysiology, Heidelberg UniversityHeidelberg, Germany; ^4^Department of Medical Cell Biology, Institute for Anatomy and Cell Biology, Heidelberg UniversityHeidelberg, Germany; ^5^Department of Molecular Neurobiology, Max Planck Institute for Medical ResearchHeidelberg, Germany

**Keywords:** glycine receptor, ivermectin, neuronal silencing, odor discrimination, rAAV

## Abstract

The causal interrogation of neuronal networks involved in specific behaviors requires the spatially and temporally controlled modulation of neuronal activity. For long-term manipulation of neuronal activity, chemogenetic tools provide a reasonable alternative to short-term optogenetic approaches. Here we show that virus mediated gene transfer of the ivermectin (IVM) activated glycine receptor mutant GlyRα_1_^AG^ can be used for the selective and reversible silencing of specific neuronal networks in mice. In the striatum, dorsal hippocampus, and olfactory bulb, GlyRα_1_^AG^ promoted IVM dependent effects in representative behavioral assays. Moreover, GlyRα_1_^AG^ mediated silencing had a strong and reversible impact on neuronal ensemble activity and c-Fos activation in the olfactory bulb. Together our results demonstrate that long-term, reversible and re-inducible neuronal silencing via GlyRα_1_^AG^ is a promising tool for the interrogation of network mechanisms underlying the control of behavior and memory formation.

## Introduction

To elucidate the mechanisms underlying cognitive processes and behavior, we need tools to reversibly manipulate neuronal activity. Classically, lesion studies have allowed researchers to study the function of certain brain regions through chronic ablation of brain tissue; however, the latter is irreversible and lacks the cellular specificity required for the analysis of neuronal networks. Therefore, novel molecular tools have been developed which permit the reversible silencing or activation of specific neurons *in vitro* and *in vivo*. In particular the field of optogenetics has expanded rapidly, and neuronal excitability can now be controlled on millisecond time scales by shining light on cells expressing either excitatory or inhibitory light-gated ion channels or pumps (Boyden et al., 2005; Zhang et al., 2007; Chow et al., 2010; Govorunova et al., 2015). However, long-term silencing and the manipulation of spatially distributed neuronal ensembles are very difficult to achieve with optogenetics (Ferenczi and Deisseroth, 2012; Raimondo et al., 2012). Hence, chemogenetic silencing receptors have been created which can negatively modulate neuronal firing rates through G_i/o_ coupled pathways after activation through pharmacologically inert drugs (Armbruster et al., 2007; Vardy et al., 2015).

Here, we tested a silencing approach, which relies on a chloride channel that is gated by the common anthelminthic drug ivermectin (IVM) (Drameh et al., 2002; Wolstenholme and Rogers, 2005) for its suitability to interrogate neuronal network function *in vivo*. As described by Lynagh and Lynch, the combination of two mutations (F207A and A288G) in the α_1_ subunit of the human glycine receptor (GlyRα_1_^AG^) strongly increases the sensitivity to nanomolar IVM concentrations of homomeric GlyRα_1_^AG^ and heteromeric GlyRα_1_^AG^β receptors with a concomitant decrease in glycine efficacy (Lynagh and Lynch, 2010). This change in ligand specificity provides the basis for IVM mediated silencing of neurons expressing GlyRα_1_^AG^. A similar approach based upon an IVM gated chloride channel from *C. elegans* (GluClαβ) has been successfully used to silence neurons *in vivo* (Lerchner et al., 2007), but relied on co-infection with two viruses, each carrying a subunit of the receptor, to be functional.

To examine whether viral GlyRα_1_^AG^ delivery can provide robust and reversible silencing of neurons in rodents, we expressed GlyRα_1_^AG^ as a T2A fusion (Tang et al., 2009) with a fluorescent reporter via recombinant adeno-associated virus (rAAV) vectors under the control of the human synapsin promotor (Kügler et al., 2003). After stereotactic virus injection, rAAV transduced GlyRα_1_^AG^ was efficiently expressed in neurons of the striatum, the hippocampus, and the olfactory bulb (OB) of mice. In these GlyRα_1_^AG^ expressing mouse cohorts, single intraperitoneal (i.p.) IVM injections evoked phenotypes in representative behavioral assays, which were fully reversible within 1 week. In the OB, we could demonstrate successful silencing of neurons at both molecular and network levels through the lack of odor induced c-Fos expression in neurons that expressed IVM sensitive GlyRα_1_^AG^, and by a reversible shift in the frequency of gamma oscillations. Our results provide strong evidence that IVM dependent long-term reversible silencing of rAAV transduced neuronal networks is a promising tool for the interrogation of network function that can be applied to various model organisms.

## Materials and methods

### Legal aspects and animal housing

All experiments were registered as biological security “level S1” at the governmental council in Tübingen, Germany (Az: 57-2/8817.40-020/MPIMF.HD.00). Animal handling and experimental procedures were performed according to the animal welfare guidelines of the Max Planck Society, and under the licenses 35-9185.81/G-71/10 and 35-9185.81/G-171/10 of the governmental council in Karlsruhe, Germany. Efforts were made to minimize numbers of animals used. Data from about 60 5–6 weeks old C57BL/6N male mice and the embryos of three pregnant female rats (Sprague-Dawley, Charles River) are presented in this study.

### Behavioral tests

C57BL/6N mice were housed on a 12-h light:12-h dark cycle (lights on at 08:00) in groups of four to six in plastic cages, with wood shavings bedding and a cardboard tube to provide some environmental enrichment. Behavioral testing was performed during the light phase. Food and water were provided *ad libitum*, except during training on appetite-motivated tasks (T-maze, olfactory discrimination task). For these, mice were fed a small amount of food pellets each day, such that their body weight was ~90% of that under free feeding conditions, but never below 80%. Before the start of a behavioral experiment, mice were handled by picking them up from their home cage and allowed to explore the experimenter's lab coat. Each mouse was handled in 5-min sessions for a minimum of 3 days before starting the experiments.

### Antibodies used in immunofluorescence (IF) and immunoblots (IB)

Primary: mα-GFP (Clontech), 1:2500–1:2000 (IB); chkα-GFP (Abcam), 1:5000 (IF); mα-NeuN (Chemicon) 1:1000 (IF); rα-2A peptide (Millipore), 1:1000 (IB); α-GAPDH (Abcam), 1:2000 (IB); rα-GFAP (Abcam), 1:700 (IF); mα-beta actin (Sigma), 1:7000 (IB); mα-4A (alpha subunits glycine receptor, Connex GmbH), 1:600 (IB), 1: 300–1:200 (IF); mα-2B (alpha 1 subunits glycine receptor (Becker et al., 1993), Connex GmbH), 1:600 (IB); mα-Beta3Tubulin (R&D Systems), 1:200 (IF); rα-cfos (Calbiochem), 1:800 (IF). Secondary: FITC-labeled α-chicken (Jackson Immuno Research), 1:700 (IF); Cy3-labeled α-mouse (Jackson Immuno Research), 1:700 (IF); Cy5-labeled α-rabbit (Jackson Immuno Research), 1:900–1:700 (IF); IRDye®700DX Conjugated (Rockland Immunochemicals), 1:7000 (IB); IRDye®800 Conjugated (Rockland Immunochemicals), 1:7000 (IB).

### Drug injections

Ivermectin (IVM, Ivomec Merial), 1.5–2.5 mg/kg, and amphetamine (AMP, Sigma Aldrich A5880), 3.5 mg/kg, were diluted in phosphate-buffered saline (PBS) for i.p. injections. The injection volume was 300 μl. Diazepam (Sigma Aldrich) was dissolved in a 10% (2-hydroxypropyl)-β-cyclodextrin solution (Sigma Aldrich) to a stock concentration of 0.4 mg/ml. 4 mg/kg diazepam were injected i.p. The injection volume was 500 μl. Control mice received PBS injections of the corresponding volume.

### Generation and production of rAAV vectors

For the generation of plasmid pAAV-Syn-GlyRα_1_^AG^-2A-Venus, the mutations F207A and A288G were introduced by primer directed mutagenesis in a GlyRα_1_ cDNA template. Next the GlyRα_1_^AG^ coding region or the non-mutated GlyRα_1_ was used to replace the ChR2A-NpHR open reading frame in pAAV-Syn-ChR2A-NpHR2A-Venus (Tang et al., 2009). For pAAV-syn-Venus, see (Pilpel et al., 2009). Viral rAAV 1/2 serotype vectors were generated in HEK293 cells as described elsewhere (McClure et al., 2011). Cells were harvested 48–60 h after transfection. Cell lysate and supernatant were pooled, and rAAVs were purified by affinity chromatography (Smith et al., 2009). Genomic titers of 10^11^–10^12^ rAAV genomes/ml virus stock were determined by Taqman RT-PCR (Applied Biosystem). Virus aliquots were stored at −80°C.

### Virus expression in hippocampal primary neurons

Dissociated hippocampal primary neurons from embryonic day 18 Sprague-Dawley rats (Charles River Laboratories) were prepared and cultured as described elsewhere (Berkel et al., 2012). About 10^4^ cells were infected with 10^8^–10^9^ viral particles 3 days after plating (DIV 3). Virus expression was monitored by fluorescence microscopy 7–21 days after infection.

### Immunofluorescence analysis of primary neurons

For immunofluorescence analysis of primary hippocampal neurons, the medium in each well was sucked away, and neurons were washed briefly with PBS at 36°C, fixed with warm 4% paraformaldehyde (PFA, 37°C) in PBS for 8 min, and washed with PBS twice. Cells were permeabilized with 0.25% Triton X-100 in PBS for 5 min, washed in PBS and blocked with 3% bovine serum albumin (BSA) and 10% normal goat serum (NGS) in PBS for 30 min prior to incubation with primary antibodies in 3% BSA for 1 h. Afterwards the cells were washed four times for 5 min in 3% BSA and stained with secondary antibodies in 3% BSA for 45 min. Then they were washed again in PBS for four times 5 min. For counterstaining 4′,6-diamidino-2-phenylindole (DAPI, Sigma-Aldrich) was added to the second washing step (1:5000). Slices were mounted on glass slides using 80% glycerol in PBS. Cover slips were fixed in place with nail polisher.

### Stereotactic injections

Stereotactic injections on 5–6 week old C57BL/6N mice were performed as described elsewhere (Cetin et al., 2006). The virus was injected via glass pipettes (tip diameter 10–20 μm). Hippocampus injection coordinates (from Bregma): −2.1 mm anteroposterior (ap), for CA1 and DG: ±1 mm lateral (lat) and 1/1.7 mm depth, for CA3: ±2.1 mm lat and 1.6 mm depth (Volume: 200–300 nl per site). Striatum: −1 mm ap, 2.2 mm lat, 3.3/3.8 mm depth (Volume: 400–450 nl). Olfactory bulb: +4 mm ap, 0.5 mm lat, 1.3/1 mm depth (Volume: 500 nl). Mice were given at least 2 weeks to recover from the surgery before behavioral testing was started.

### Electrophysiology in acute hippocampal slices

Mice were injected unilaterally with rAAV-syn-GlyRα_1_^AG^-2A-Venus or rAAV-Syn-Venus (stereotactic injection coordinates: CA3: −2.1 mm ap., ±2.1 mm lat., and 1.6 mm depth; Volume: 300–350 nl per site). Transverse hippocampal slices (300 μm thick) were prepared 10–14 days after virus injection. The slicing chamber contained an oxygenated ice-cold solution (modified from Dugué et al., 2005), composed of (in mM): K-gluconate, 140; N-(2-hydroxyethyl) piperazine-N′-ethanesulfonic acid (HEPES), 10; Na-gluconate, 15; ethylene glycol-bis (2-aminoethyl)-N,N,N′,N′-tetraacetic acid (EGTA), 0.2; and NaCl, 4 (pH 7.2). Slices were incubated for 30 min at 35°C before being stored at room temperature (RT) in artificial CSF (ACSF) containing (in mM): NaCl, 125; NaHCO_3_, 25; KCl, 2.5; NaH_2_PO_4_, 1.25; MgCl_2_, 1; CaCl_2_, 2; and D-glucose, 25; bubbled with 95%O_2_ and 5%CO_2_. During experiments, slices were continuously perfused with ACSF. Patch electrodes were pulled from hard borosilicate capillary glass (Sutter Instruments flaming/brown micropipette puller). Electrodes for the voltage clamp experiment were filled with a high chloride solution which consisted of (in mM) Cs-gluconate, 105; CsCl, 30; HEPES, 10; MgATP, 4; MgGTP, 0.3; phosphocreatine, 10 (pH 7.3 with CsOH). The low chloride solution for current clamp experiments consisted of (in mM) K-gluconate, 130; HEPES, 10; KCl 4; MgATP, 4; MgGTP, 0.3; phosphocreatine, 10 (pH 7.3 with KOH). For registration of spiking activity in the cell-attached mode, electrodes were filled with ACSF. CA3 pyramidal cells were identified visually using IR-video microscopy. Whole-cell recordings from these neurons were taken at 32°C using a HEKA EPC-7 amplifier (List Elektronik) with a sampling rate of 100 μs and filtered at 3 kHz.

### Immunostaining of fixed brain slices

Mice were transcardially perfused with PBS followed by 4% Paraformaldehyde (PFA) in PBS. Brains were post-fixed for 12 h at 4°C. Free-floating sections (70 μm) were cut using a vibratome (Leica). For cryostat cutting (c-Fos staining protocol), brains were immersed in 20% sucrose in PBS overnight followed by 30% sucrose in PBS overnight. Afterwards, brains were frozen in a small glass beaker filled with isopentane embedded in dry ice. After 2 min in isopentane, frozen brains were stored at −80°C. Before sectioning, the frozen brains were transferred for 3–4 h to −20°C and then cut in 50 μm slices on a cryostat (Mikrotom Bright Kryostat Modell OTF, Hater instruments). All sections were stained free-floating. Vibratome and cryostat slices were incubated for 1 h at room temperature (RT) in blocking buffer (2% gelatine, 2% BSA, 0.1% Triton X-100 in PBS). Primary antibodies diluted in blocking solution were added for an over-night incubation at RT. After washing with a 1:3 dilution of blocking buffer, slices were transferred to fresh blocking buffer containing fluorophore-conjugated secondary antibodies for 1.5 h at RT. In some cases DAPI (1:5000) was mixed into the final washing steps. After a final wash with PBS, slices were mounted on glass slides using 80% glycerol in PBS. Cover slips were fixed in place with nail polisher.

### Image acquisition

Wide-field fluorescence images were acquired with a Zeiss Axioimager.M1 (Carl Zeiss). Confocal images were acquired at a resolution of 1024 × 1024 pixels with a Leica SP2 (Leica) microscope equipped with 10× to 63× (glycerol immersion) objectives, an UV laser (352 nm), an Argon laser (450–530 nm), and two Helium Neon lasers (543 nm). Acquired confocal images were loaded into ImageJ (Rasband, W.S., ImageJ, U. S. National Institutes of Health, Bethesda, Maryland, USA, http://imagej.nih.gov/ij/, 1997–2015), and a maximum intensity projection of 4–6 adjacent slices was calculated. c-Fos immunofluorescence in the OB: A software controlled x-y stage controller of the Leica SP2 confocal laser scanning microscope was used to scan the whole brain slice, and for every slice at least 4 depths (z) were imaged. After acquisition, the images were stitched together using custom Matlab (Mathworks) routines. The stitched image, which had the most homogenous αNeuN signal throughout, was chosen for further analysis. Six to thirteen 150 × 150 μm square regions were counted per slice (one slice per mouse).

### Expression analysis

For the analysis of virus expression patterns, wide-field fluorescence images of αGFP (Venus) stained slices of matching anterior-posterior coordinates were first aligned to each other and to schemata taken from a mouse brain atlas (The Mouse Brain in Stereotaxic Coordinates, George Paxinos and Keith B. J. Franklin, Academic Press). Aligned images were then loaded into ImageJ, and the same gamma correction was applied to all images to decrease background fluorescence and enhance signal visibility. An average intensity projection was then calculated, and the contrast was automatically enhanced (ImageJ; Enhance Contrast: Saturated pixels: 1%). A perceptually uniform sequential colormap was then applied for pseudo-coloring (https://github.com/BIDS/colormap, option D).

### Immunoblot analysis

Primary hippocampal cultures (14 days after infection) were harvested with lysis buffer (80 μl/well; 10 mM Tris pH 7.4, 150 mM NaCl, 2 mM EDTA, 0.5% NP40, 1% TX-100). For sample extraction from brain tissue, mice were killed and decapitated and their brains dissected on ice. A sub region of the striatum that was targeted by the stereotactic injection was extracted and homogenized with 600 μl ice-cold Buffer (0.32 M sucrose, 2 mM EDTA, 5 mM HEPES pH 7.4). All preparations were supplemented with a protease inhibitor cocktail (CompleteTM; Roche). Protein concentrations were measured using the Bradford reagent (Sigma Aldrich). Cell lysates were separated via SDS-polyacrylamide gel electrophoresis (10% separating and 4% stacking gels) and transferred to nitrocellulose membranes in an electrophoresis chamber over night. The membranes were blocked with 8% non-fat dry milk in PBS with 0.05% Tween® 20 (PBS-T) for 1.5 h at room temperature (RT) and were incubated with primary antibodies overnight at 4°C. On the next day the membranes were washed three times with PBS-T for 20 min and then treated with the secondary antibodies in PBS-T for 1.5 h at RT. After three final washing steps with PBS-T for 20 min the membranes were analyzed on a FLA9000 fluorescence scanner (GE Healthcare, Life Sciences).

### Rotational bias testing

Mice were placed in a round metal kitchen bowl (30 cm diameter) spray painted in white. Videos were recorded from top for 45 min and analyzed offline (Webcam, Logitech, 640 × 480 pixels). All videos were analyzed using custom Matlab (Mathworks) routines. Briefly, every analyzed frame of the video (30 fps original video acquisition, actual analysis rate 5 fps) was thresholded, and a series of morphological operations was applied to confine the boundaries of the extracted pixel region to the actual mouse body. The 3-point extraction of the mouse body was achieved by initializing a k-means clustering algorithm (*k* = 3) of the extracted mouse body pixel region with a slightly larger region that included the mouse body and tail. This permitted the subsequent extraction of three center points (front part center, center of the whole region, back part center), and thereby the extraction of a rotation angle. The extraction was robust over a wide range of imaging conditions, as controlled manually in a subset of the analyzed videos (see also Supplementary Movie 1). All recorded rotation angles were saved, and differences between rotation angles of subsequent frames were filtered in between 4 and 120°. Both boundaries were set empirically after manual evaluation of the analysis process had shown that angles below 4° represented mostly noise, and that angles above 120° were not occurring naturally, but represented errors in which the head-tail direction was accidentally reversed. This way also baseline crossings (0°) were filtered out which facilitated the analysis. Rotational bias was calculated as relative amount of left over all turns. The difference to baseline (Δ Rotation) is given as modulus|(bias after IVM-baseline bias)|.

### Rotarod

Motor-coordination of mice was analyzed on the Rotarod treadmill (Ugo Basile). The Rotarod consists of a rotating drum (30 × 3 cm) suitably machined to provide grip. Six flanges divide the drum in five 5.7 cm wide lanes, enabling 5 mice to be analyzed simultaneously. The mice were put on the rotating drum at a rotation speed of 4 rpm. For each trial, the speed of rotation increased from 4 to 40 rpm within 8 min. The time to fall off the drum (latency) was recorded for each mouse.

### Open field and novel object exploration

Mice were allowed to explore an open field (50 × 50 × 30 cm) in a brightly lit environment. After 10 min, an object (children toy) was inserted in the center of the open field (novel object exploration phase), and mice were allowed to explore object and open field for another 5 min. The mouse path was digitized with a tracing software (TSE Systems GmbH) and stored in a text file, which allowed to extract the percentage of time spent in different regions of the open field and the activity (total path length) for each mouse.

### T-maze

The rewarded version of the T-maze was performed with fasted mice as described (Deacon and Rawlins, 2006). In brief: For habituation, each animal was placed on the start arm of the T-Maze, with embedding material of the home cage scattered on the maze. After 30 s, the block of the start arm was removed, and the mice were given 3 min to find and eat the two food pellets (TSE Dustless Precision Pellets, TSE Systems GmbH) in the two goal arms. Then the mice were put back into their home cages. The actual T-Maze test (rewarded non-matching to sample) always started with placing the mouse on the start arm. In the forced sample trial, the entry to one of the goal arms was randomly blocked. The mouse was allowed to explore the maze and to eat the food pellet deposited in the open arm. For the subsequent free choice trial, the mouse was placed back on the start arm, and the block to the second goal arm was removed. If the animal entered the arm that had been previously blocked in the forced sample trial, this was counted as a correct decision. However, if the animal re-entered the previously unblocked arm, this was counted as wrong decision. The minimum inter-trial interval for each mouse was about 45 min. On each test day, 8 trials per mouse were analyzed. During the course of this experiment, one virus uninjected control mouse was excluded from final analysis, since it showed erratic behavior and dropped to 25% performance on the third test day.

### Pellet seeking task

For the pellet seeking task, the mouse was transferred to a novel cage in which a food pellet (TSE Dustless Precision Pellets, TSE Systems GmbH) was hidden under the wood shavings. The time to find the pellet in the cage was measured. Unsuccessful trials were terminated after 10 min. Search times were determined in two subsequent trials; tests were performed twice for each mouse: prior to and 1.5 days after i.p. injection of 2.5 mg/kg IVM.

### Olfactory discrimination task

The olfactory discrimination procedure was adapted from Mihalick et al. (2000). Fasted mice (80–90% original body weight) were tested in their home cage, which was placed under a fume hood during all tests. The home cage was split into an empty home compartment and a test compartment by a plastic wall with a door at its center. The rewarded test compartment contained a removable plastic rig, which held two sand-filled lids in place. In the training phase before the actual discrimination task (4 days, 4 trials per mouse per day), mice were presented with only one sand filled, unscented lid per trial that was randomly placed on the left or right side on subsequent trials. In the first 2 days, mice had to discover 3 food pellets (one uncovered on top of one lid, two other buried in the sand of the lid), and consume them within 15 min. Over the next 2 days, only one pellet was hidden in the sand filled lid, and mice had 15 min to find the reward. During the training, mice were also getting accustomed to sand filled lids that were placed in their home cage overnight. In the second phase, the actual discrimination training, mice learned to associate an odor, either 2-phenylethanol or vanillin (both Sigma Aldrich) to the location of a food pellet. 2-phenylethanol and vanillin were chosen since it is known that they are pure odors and therefore processed exclusively by the main olfactory bulb (Doty et al., 1978; Frasnelli et al., 2010). Care was taken that the same position was not chosen more than 3 times on consecutive trials. Odor dilutions (2% in water) were freshly prepared on every day. At the beginning of every trial the sand was completely renewed, a food pellet was put in each of the lids, and 100 μl of diluted odor solution was distributed on top of the sand. Moreover, each mouse had its own set of lids that was not used for testing or training other mice. A rewarded/unrewarded odor pair was randomly assigned to each mouse. The position of each scented lid was randomized for each trial. In each trial (8 per day, 5 min maximum, see also Supplementary Movie 2), the mouse was first placed in the home compartment, and the rig with one rewarded and one non-rewarded odor lid was put in the test compartment. After 20 s, the home compartment door was opened and the mouse could enter the test compartment. Searching in the sand with the right odor was rewarded with a food pellet. The time was registered until digging began in one lid (Supplementary Figure 7, “Time to decision in discrimination task”). If mice started to dig in the lid with the non-rewarded odor, the trial was ended unrewarded with the retraction of the entire rig. Inter-trial intervals were at least 30 min. If mice performed at or above a threshold criterion of 7 out of 8 correct trials per day on at least 2 consecutive days, they were i.p. injected with 2.5 mg/kg IVM and were tested again after 0.5 and 1, 5 days. Then testing was interrupted for the next 7 days, and mice were re-tested on days 8 and 9.

### c-Fos induction protocol

Unilaterally injected mice were randomly assigned to two groups, of which one received IVM (2.5 mg/kg) i.p., while the other was injected with PBS i.p. only. Twenty hours after the i.p. injections, mice were stimulated intermittently with a mixture of odors and clean air under a fume hood (Inaki et al., 2002). For this, mice were transferred to the test room 3 h prior to the odor induction protocol. Each mouse was placed subsequently into two 2 l glass beakers for 5 min. First in a covered beaker that contained filter paper scented with 2-phenylethanol, vanillin, and wood shavings from the cages of other mice and then in an open empty beaker, again for 5 min. This cycle was repeated 3 times for each mouse, and then the mouse was transferred to its home cage and killed 1 h later for immunohistological analysis.

### Intracranial local field potential (LFP) recordings in the olfactory bulb

Stereotrodes were manufactured from 50 μm thick, isolated tungsten wire glued together with a fast drying adhesive (Roti Coll 1, Carl Roth GmbH). Mice were prepared for surgery as described for rAAV injections, but continuous isoflurane anesthesia (2.5–3%) instead of Ketamine/Xylazine was used during the whole surgery. The same stereotactic coordinates as for the rAAV injections into the OB were used. The tungsten electrodes were carefully lowered into the olfactory bulb and fixed with dental cement (Hager and Werken, Cyano Veneer). They were then lead and fixed to a custom-made head stage (similar to EIB-16, Neuralynx) that held the connector necessary for recordings (Omnetics Connector Corp, A79042-001). A bare metal screw inserted over the cerebellum acted as combined reference and ground electrode for the pre-amplifier. The whole assembly was held in place with dental cement. The skin was sutured where necessary. Directly after the surgery and 12/24 h afterwards, mice were i.p. injected with 5 mg/kg carprofen (Rimadyl, Pfizer) as post-surgical analgesic (Adamson et al., 2010). Mice were given 1 week for recovery. For intracranial LFP recordings, a 16 channel pre-amplifier board (RHA 2116, Intan technologies) was connected to the head stage to which all 4 electrodes (2 stereotrodes) were connected. Pre-amplified signals were digitized on a laptop at 25 kHz (RHA2000-EVAL USB interface board, Intan Technologies) and saved for offline analysis without prior filtering. In every session, 10 min periods were sampled two times while mice were exploring an open field covered with wood shavings. Videos of their behavior were recorded from the top and analyzed offline (Webcam, Logitech, 640 × 480 pixels) by tracing the position of the animals with routines written in Matlab (Mathworks), which allowed to extract the total path length covered (Supplementary Figure 5B). After recording, animals were deeply anesthetized with ketamine/xylazine, and electrical lesions were induced twice (20 μA, 10 s) for each single tungsten wire separately to clearly mark electrode positions.

### Offline analysis of LFP recordings

The raw LFP signal was analyzed offline via custom Matlab (Mathworks) routines. Briefly, the power spectra of every 10 min recording were extracted via a power spectral density estimate (Matlab: “pwelch,” 4 s Hamming window, 50% overlap). A linear fit was then subtracted from the power spectra (Matlab: “detrend”), and the gamma peak frequency was found in between 50 and 90 Hz. No further down sampling or other corrective routines were employed. Signals from the left and right side of the OB were interpreted as independent samples, and signals from both electrodes of the stereotrode on one side were averaged. Since effects on gamma peak frequency could differ in between both halves of the bulb, depending on the spread of virus expression and the exact position of the electrodes, signals from the left and the right side were sorted for their difference to the baseline gamma peak frequency 0.5 days after IVM injection in both GlyRα_1_^AG^ expressing and Sham (PBS) injected mice (Supplementary Figure 5 “Sorted: weak” and “Sorted: strong”). For the insets in **Figure 4C**, spectral density estimates were smoothed in Adobe Illustrator 17.0.0 (Object > Path > Simplify).

### Statistical analysis

**Figure 1:**
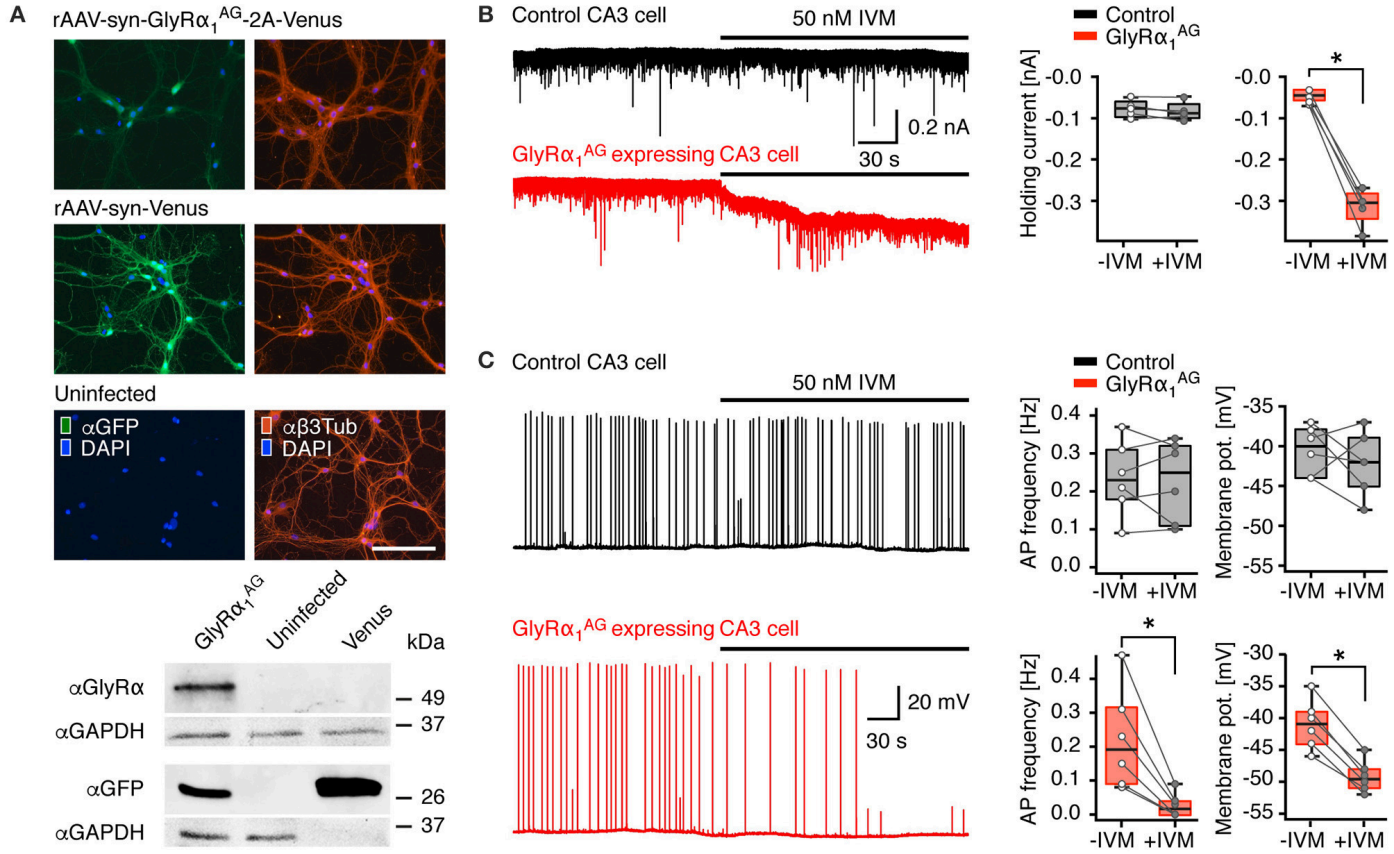
**GlyRα_**1**_^**AG**^ expression in transduced hippocampal primary neurons and acute slices of virus injected mice. (A)** Top: Rat hippocampal primary neurons infected with rAAV-syn-GlyRα_1_^AG^-2A-Venus, rAAV-syn-Venus, and uninfected control; left column: Venus fluorescence amplified with an anti-GFP immunostain (αGFP, green); right column anti-Beta3Tubulin (αβ3Tub, red). The rAAV-syn-GlyRα_1_^AG^-2A-Venus infected cells show regular morphology and branching compared to rAAV-syn-Venus infected and uninfected cells; nuclei stained with DAPI (blue); scale bar 125 μm. Bottom: Cut outs of αGlyRα, αGFP and αGAPDH immunoblots of primary neuron cell extracts infected with rAAV-syn-GlyRα_1_^AG^-2A-Venus (GlyRα_1_^AG^), rAAV-syn-Venus (Venus), and uninfected cells. Please note that for αGFP blots the protein amount of rAAV-syn-Venus infected cell extracts loaded was 1 μg as compared to 10 μg for all other lanes shown. The full version of the immunoblot is presented in Supplementary Figure 1. **(B)** Patch clamp recordings from acute brain slices of mice injected with either rAAV-syn-GlyRα_1_^AG^-2A-Venus or rAAV-syn-Venus (control) into the hippocampus. IVM application failed to trigger any detectable current in CA3 pyramidal cells in slices from control animals (black trace), but activated a strong outward chloride current in Venus positive cells in slices from rAAV-syn-GlyRα_1_^AG^-2A-Venus injected animals (red trace); box plots (right) show cumulative data from *n* = 5 cells each (median, 25th/75th percentile); ^*^p < 0.05 **(C)** IVM bath application had no effect on the AP frequency or the membrane potential of control neurons (representative black trace) but lead to a strong decrease in AP frequency and membrane potential in GlyRα_1_^AG^ expressing CA3 pyramidal cells (representative red trace); box plots on the right summarize data from all recorded neurons (median, 25th/75th percentile); ^*^p < 0.05. The open and filled circles represent AP frequency and membrane potential in individual experiments before and after IVM application (*n* = 6, except membrane potential of controls with *n* = 5), respectively.

All statistics except the hippocampal slice electrophysiology experiments (Figures 1B,C; Supplementary Figure 2, Wilcoxon Rank Sum Test) were calculated in GraphPad Prism. Non-parametric test statistics were used where normal distribution of analysis results could not be proven. The level of significance was set at *p* < 0.05.

## Results

### Viral transduction of GlyRα_1_^AG^ leads to robust expression in primary neurons

The efficiency of rAAV-syn-GlyRα_1_^AG^-2A-Venus, which encodes GlyRα_1_^AG^ linked to the fluorescent reporter protein Venus via the T2A peptide bridge (Tang et al., 2009; Supplementary Figure 1, top), was tested in hippocampal primary neurons. Two weeks after rAAV infection, virus-infected cells could be visualized by enhanced Venus fluorescence (Figure 1A, top; Supplementary Figure 1A, bottom, αGFP), and expression of GlyRα_1_^AG^ was confirmed by staining with αGlyRα (Supplementary Figure 1A, bottom, αGlyRα). GlyRα_1_^AG^-2A-Venus expressing cells showed regular morphology, and αβ3Tubulin staining did not reveal any difference in neurite growth between rAAV-syn-GlyRα_1_^AG^-2A-Venus, rAAV-syn-Venus infected and uninfected primary neurons (Figure 1A). Venus fluorescence was more intense in rAAV-syn-Venus than in rAAV-syn-GlyRα_1_^AG^-2A-Venus infected neurons, and immunoblots showed that Venus protein expression in the rAAV-syn-Venus infected neurons was at least 10 times stronger than in cells infected with rAAV-syn-GlyRα_1_^AG^-2A-Venus (Figure 1A, bottom; full membrane shown in Supplementary Figure 1B). The presence of immunoreactive GlyRα was only detectable in extracts of rAAV-syn-GlyRα_1_^AG^-2A-Venus infected neurons (Figure 1A) at a slightly higher apparent molecular weight than GlyRα_1_^AG^ (48 kDa) due to the adherent C-terminal 2A peptide (Supplementary Figure 1); notably, no 2A-fusion proteins were detectable (Supplementary Figure 1B), indicating that the lower expression of rAAV-syn-GlyRα_1_^AG^-2A-Venus is not due to incomplete ribosomal skipping at the T2A peptide bridge.

### GlyRα_1_^AG^ expression in hippocampal neurons leads to IVM dependent silencing of neuronal activity

To test the silencing potential of virally expressed GlyRα_1_^AG^, we compared the activity pattern of hippocampal pyramidal cells in acute brain slices prepared from mice that had received unilateral hippocampal injections of either rAAV-syn-GlyRα_1_^AG^-2A-Venus or rAAV-syn-Venus (controls). Infected CA3 pyramidal cells were identified by Venus fluorescence and patched with a high chloride (30 mM) cesium containing intracellular solution. Cells were held at −70 mV. After obtaining a stable base line, 50 nM IVM was bath-applied. In slices from control animals, IVM did not cause significant changes in the holding current measured before (−83 [−95.5/−62.5] pA; median [25th/75th percentile]) and after (−89.5 [−102.5/−61.5] pA) drug application (Figure 1B; *n* = 5; *p* > 0.05). However, in neurons of rAAV-syn-GlyRα_1_^AG^-2A-Venus infected mice, IVM administration resulted in a strong and significant increase from −58.5 [−69.5/−40.5] pA to −299 [−343.5/−282.5] pA (Figure 1B; *n* = 5; ^*^p < 0.05). Thus, IVM activated a strong non-desensitizing current in GlyRα_1_^AG^ expressing neurons. In order to determine whether activation of virally expressed GlyRα_1_^AG^ can significantly reduce neuronal excitability, we conducted a set of experiments in which the spontaneous firing of CA3 pyramidal cells was enhanced by both blocking GABA_*A*_-mediated inhibition (10 mM Gabazine) and elevating the extracellular potassium concentration (6 mM K^+^); recordings were conducted in the current clamp mode using an intracellular solution containing 4 mM chloride. Under these conditions, CA3 pyramidal cells were firing single action potentials (APs) or short bursts of 2–5 APs with an average frequency of around 0.2 Hz in both control and GlyRα_1_^AG^ expressing slices. Application of IVM did not change the firing rate (Figure 1C top; −IVM: 0.23 [0.18/0.31] Hz, +IVM: 0.25 [0.11/0.32] Hz; *n* = 6; *p* > 0.05) or the resting membrane potential (−IVM: −40 [−38/−44] mV, +IVM −42 [−39/−45] mV; *n* = 5; *p* > 0.05) of CA3 pyramidal cells in rAAV-syn-Venus injected animals. However, in GlyRα_1_^AG^ expressing cells IVM administration led to a significant drop in firing frequency (Figure 1C bottom; −IVM: 0.19 [0.09/0.31] Hz, +IVM: 0.02 [0/0.04] Hz; *n* = 6; ^*^p < 0.05) and hyperpolarization (from −41 [−44/−39] mV to −49 [−51/−48] mV; *n* = 6; ^*^p < 0.05). Since in the whole cell configuration the effect of IVM triggered GlyRα_1_^AG^ activation depends on the intracellular chloride concentration, we tried to further substantiate these results with a set of experiments, in which APs were detected in the cell-attached configuration. Again, the excitability of CA3 pyramidal cells was found to increase upon the elevation of extracellular potassium concentration and gabazine addition. On average, the firing rates recorded in the cell-attached configuration were higher (about 0.5 Hz) as compared to those obtained in the whole-cell mode. In slices from control animals, the application of IVM failed to reduce firing frequencies (Supplementary Figure 2, top; −IVM: 0.55 [0.3/0.75] Hz, +IVM: 0.63 [0.39/0.68] Hz; *n* = 5; *p* > 0.05). In contrast, in neurons expressing GlyRα_1_^AG^ IVM caused robust reductions of the firing rate from 0.41 [0.26/0.7] Hz to 0.03 [0/0.065] Hz (Supplementary Figure 2, bottom; *n* = 5; ^*^p < 0.05).

### Hippocampal expression of GlyRα_1_^AG^ leads to IVM dependent hyperactivity and deficits in spatial working memory

**Figure 2:**
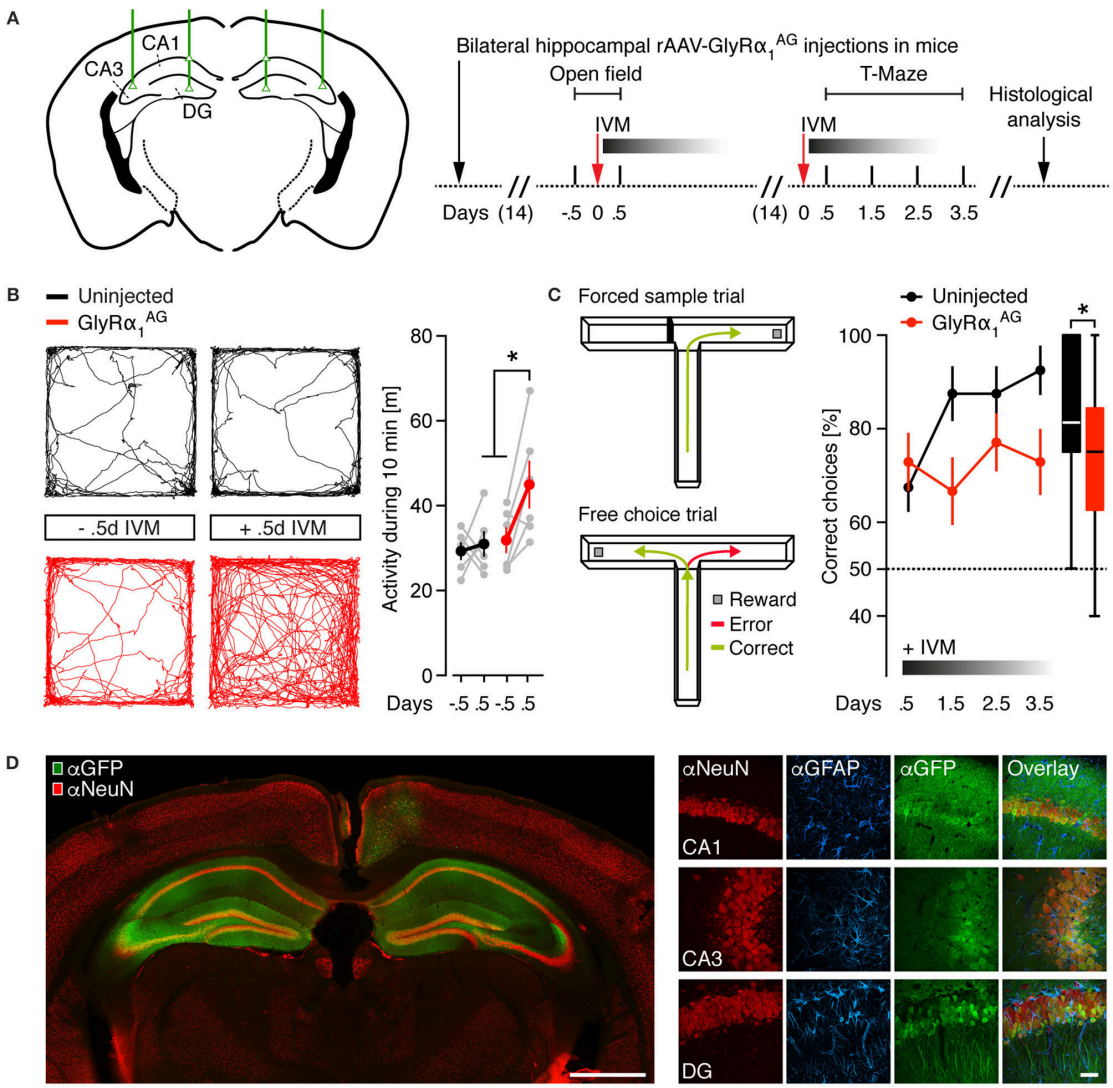
**IVM induced hyperactivity and spatial working memory impairment in mice with bilateral neuronal expression of GlyRα_**1**_^**AG**^ in the dorsal hippocampus**. **(A)** Left: Injection scheme, green lines and triangles indicate the coordinates used for bilateral stereotactic rAAV-syn-GlyRα_1_^AG^-2A-Venus injections into the dorsal hippocampus of mice (DG, dentate gyrus; CA1 and CA3, cornu ammonis 1 and 3). Right: Time line of the experiment. **(B)** Left: Representative trace diagrams in an open field of one virus uninjected control and one GlyRα_1_^AG^ transduced mouse before (−0.5 d) and after (+0.5 d) IVM exposure. Right: GlyRα_1_^AG^ transduced mice showed a hyperactive phenotype 0.5 days after the i.p. injection of IVM; GlyRα_1_^AG^ *n* = 6, Uninjected *n* = 6; data shown as means ± SEM; ^*^p < 0.05. **(C)** Left: The two phases of the T-Maze rewarded alternation task: One arm is randomly blocked in the first trial (Forced sample trial) and re-opened in the second trial (Free choice trial); alternations in the free choice trial are counted as correct, while re-entries into the same arm as entered in the forced sample trial are counted as error. Right: 0.5–3.5 days after IVM injection, GlyRα_1_^AG^ expressing mice performed worse than virus uninjected control mice in the rewarded alternation task; GlyRα_1_^AG^ *n* = 6, Uninjected *n* = 5; data shown as means ± SEM; box plot on the right shows cumulative data over all days and trials (median, 25th/75th percentile, ^*^p < 0.05). **(D)** Left: Representative image of a coronal brain section from one rAAV-syn-GlyRα_1_^AG^-2A-Venus injected mouse [αNeuN (red) and αGFP (green) staining]; Venus expression is prominent in both hippocampi; scale bar: 1 mm. Right: Confocal imaging (maximum intensity projections) of hippocampal subregions in coronal brain slices from a rAAV-syn-GlyRα_1_^AG^-2A-Venus injected mouse, immunostained for αNeuN (red), αGFAP (blue), and αGFP (green); note colocalization of NeuN and GFP fluorescences; scale bar: 20 μm.

Recently, it has been shown that the trial-restricted silencing of hippocampal excitatory neurons leads to short- and long-term memory deficits in spatial learning (Shipton et al., 2014). Since IVM delivery activates GlyRα_1_^AG^ over several days, we speculated that GlyRα_1_^AG^ mediated silencing might be an excellent tool for the investigation of multi-trial based experiments. We therefore examined whether the activation of hippocampally expressed GlyRα_1_^AG^ by IVM results in an impairment of spatial working memory (SWM), which is hippocampus dependent and tested over several days (Deacon and Rawlins, 2006). Two weeks after bilateral hippocampal rAAV-syn-GlyRα_1_^AG^-2A-Venus injection (Figure 2A), GlyRα_1_^AG^ transduced mice showed regular Rotarod performance compared to virus uninjected control mice, and we found no differences between groups before or 0.5 days after the injection of 2.5 mg/kg IVM in a novel object exploration test (Supplementary Figures 3A–C, GlyRα_1_^AG^ *n* = 6, uninjected *n* = 6). However, 0.5 days after the delivery of IVM, GlyRα_1_^AG^ expressing mice showed higher activity levels (total path length) during 10 min of free exploration in an open field (Figure 2B, GlyRα_1_^AG^ *n* = 6, uninjected *n* = 6; ^*^p = 0.028, Paired *t*-test, two-tailed) and compared to uninjected mice after IVM delivery (Figure 2B, GlyRα_1_^AG^ *n* = 6, uninjected *n* = 6; ^*^p = 0.026, Mann Whitney test). This finding is in line with other studies, which demonstrated that animals become hyperactive after lesions to the dorsal or ventral hippocampus (Douglas and Isaacson, 1964; Nadel, 1968).

Mice were then given 2 weeks to recover from the first IVM injections before SWM was analyzed in the food rewarded version of the T-Maze task (Deacon and Rawlins, 2006). For this experiment, mice were first starved to <90% of their initial body weight (Supplementary Figure 3D). In the T-maze test, the starved mice are initially forced by a physical block to enter either the right or left arm of the maze to receive a food reward (forced sample trial). In the following free choice trial, both arms are open but only the previously unvisited arm is rewarded. During the free choice trial, an entry in the previously blocked arm is counted as an error (Figure 2C, left). All mice were injected once with 2.5 mg/kg IVM, and 0.5 days later the first block of eight trials was performed. As shown in Figure 2C, right, in contrast to virus uninjected mice, GlyRα_1_^AG^ transduced animals did not show any improvement during four subsequent test blocks (Figure 2C, GlyRα_1_^AG^ *n* = 6, uninjected *n* = 5; ^*^p = 0.0181, performance over all days, Mann Whitney test), but still performed significantly better than chance level (One sample *t*-test to a theoretical mean of 50%: GlyRα_1_^AG^: ^*^p = 0.0019, uninjected: ^*^p = 0.0089). This is consistent with IVM injection causing a mild SWM impairment in GlyRα_1_^AG^ expressing mice. In all rAAV injected animals, a wide-spread expression of the fluorescent reporter was observed in both dorsal hippocampal formations (Figure 2D). Furthermore, in a neuronal (αNeuN) and glial [glial fibrillary acidic protein (αGFAP)] immunostain of the hippocampus, the rAAV injected animals showed neuron-specific expression of GlyRα_1_^AG^ and no signs of scar tissue formation (Figure 2D).

Together these results show that the bilateral IVM mediated activation of GlyRα_1_^AG^ in the hippocampus of mice leads to hyperactivity and deficits in a SWM paradigm 0.5 days after drug delivery.

### Unilateral striatal expression of GlyRα_1_^AG^ leads to an IVM dependent rotational phenotype

**Figure 3:**
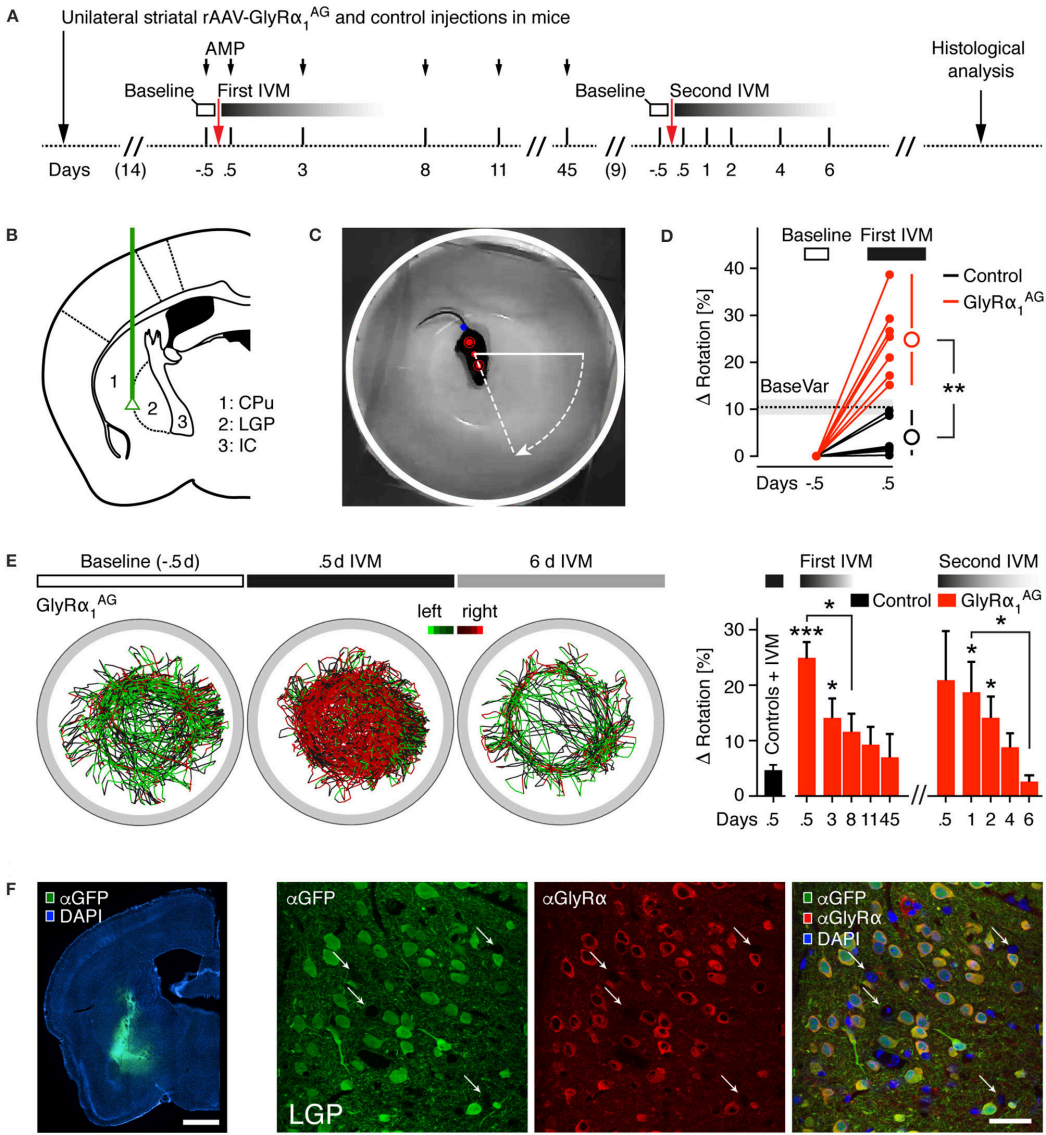
**Unilateral expression of GlyRα_**1**_AG in the striatum of mice leads to a rotational phenotype after intraperitoneal IVM injection. (A)** Time line of the experiment (AMP, amphetamine; IVM, ivermectin); injection time points of IVM are shown as arrows. **(B)** Injection sites; green line indicates path of injection cannula for unilateral striatal rAAV-syn-GlyRα_1_^AG^-2A-Venus and mock control (rAAV-syn-GlyRα_1_-2A-Venus and rAAV-syn-Venus) injections. Subregions depicted: CPu, caudate putamen; LGP, lateral globus pallidus; IC, internal capsule. **(C)** Camera picture and overlay showing the automatic extraction of three points on the mouse body (red dots) used to trace the rotation angle; tail point is marked in blue. **(D)** The baseline variability (BaseVar) of the rotational bias was quantified for all control mice on trials without preceding IVM injection; the i.p. injection of IVM led to a strong increase in rotational bias in all GlyRα_1_^AG^ expressing mice 0.5 days after IVM exposure as compared to IVM treated, mock injected control animals; open circles indicate group averages (ΔRotation: Difference to baseline bias). **(E)** Left: Example trace diagrams of a mouse injected with rAAV-syn-GlyRα_1_^AG^-2A-Venus in the left hemisphere (45 min per trace), color coded for left (green) and right (red) turns; 0.5 days after IVM injection a strong bias to the right was found, which reversed after 6 days. Right: Time course of rotational bias after the first (2.5 mg/kg) and second (1.5 mg/kg) IVM injections in GlyRα_1_^AG^ expressing mice; first bar on the left (black) shows all mock injected control mice 0.5 days after IVM injection (Controls + IVM, pooled first and second IVM injection); stars on top of bars indicate statistical significance vs. Controls + IVM (first bar on the left); 3.5 mg/kg AMP was injected i.p. 10 min before the recording started (First IVM). First IVM: GlyRα_1_^AG^ *n* = 7, Control *n* = 6; Second IVM: GlyRα_1_^AG^ *n* = 5; Control *n* = 5; data are shown as means ± SEM; ^*^p < 0.05, ^**^p < 0.01, ^***^p < 0.001. **(F)** Left: Representative image of a coronal brain section of a rAAV-syn-GlyRα_1_^AG^-2A-Venus injected mouse, immunostained for Venus (αGFP, green) and counterstained with DAPI (blue); scale bar 1 mm. Right: Maximum intensity projections of confocal microscopy images acquired in a brain slice in the injected region (LGP), immunostained for Venus (αGFP, green) and GlyRα_1_^AG^ (αGlyRα, red), counterstained with DAPI (blue). Note GlyRα staining surrounding Venus expressing neurons; white arrows indicate putative uninfected neurons; scale bar: 40 μm.

Next, we monitored the rotation behavior in mice with unilateral striatal GlyRα_1_^AG^ expression. This paradigm allows for the robust behavioral readout of striatal lesions upon induction with amphetamine (AMP) (Mandel and Randall, 1985) and can be used to demonstrate the reversibility of IVM mediated silencing (Lerchner et al., 2007; Hu et al., 2014). For this test, we employed seven mice that received unilateral striatal injections of rAAV-syn-GlyRα_1_^AG^-2A-Venus and six mock-injected mice as controls (rAAV-syn-GlyRα_1_-2A-Venus, *n* = 3, and rAAV-syn-Venus, *n* = 3) (Figures 3A,B). Two weeks post injection, all animals were tested for rotational bias in a small, round kitchen bowl, which makes the quantification of subtle turning biases feasible (Barber et al., 1973; Jerussi, 1982) (see Section Materials and Methods). In total, 141 videos of 45 min length were analyzed. To facilitate the analysis of rotational biases, an algorithm was developed which automatically extracts the body angle for every frame in the recorded video (Figure 3C; Supplementary Movie 1). This way, left and right turns could be quantified reliably over time, which then allowed calculating a turning bias (turns to one side/all turns) for every trial. A baseline bias that was determined from the trial prior to IVM injection was subtracted for each mouse. As shown in Figure 3D (“BaseVar”), the overall variability in between trials for controls without preceding IVM injection was 10.4 ± 1.43% (Mean ± SEM, 43 videos of 6 control mice). The first test trial was performed 0.5 days after drug injection, since it is known that IVM reaches its peak behavioral effect after this time (Lerchner et al., 2007) and our own preliminary results had confirmed this observation (data not shown). In the first set of experiments (“First IVM”), mice were injected with 3.5 mg/kg AMP 10 min before the rotation behavior was recorded.

As demonstrated in Figure 3D (“First IVM”), 0.5 days after the injection of 2.5 mg/kg IVM, GlyRα_1_^AG^ transduced mice showed a strong change in rotational bias (24.79 ± 3.02%, Mean ± SEM, *n* = 7), and the difference between GlyRα_1_^AG^ and control mice reached high statistical significance (^**^p = 0.0012, Mann Whitney test). Control mice also showed rotational biases, however only within baseline variability (3.96 ± 1.68%, Mean ± SEM, *n* = 6). Mice were re-tested on subsequent days (see representative trace diagram of one GlyRα_1_^AG^ expressing mouse in Figure 3E), and the complete time course of the rotational bias is shown in Figure 3E on the right. Interestingly, the IVM elicited phenotype was reversible over the following days, such that the difference between GlyRα_1_^AG^ expressing and control mice (Controls + IVM) was still significant after 3 days (^*^p = 0.012, Mann Whitney test), but dropped below significance on day 8 (*p* = 0.057, Mann Whitney test). It declined further until the last time point examined, i.e., 45 days after the first IVM injection. Some mice were killed for analysis after the first IVM injections, and others were re-injected with IVM at a slightly lower concentration (1.5 mg/kg) 9 days afterwards. The testing in this second round of injections was performed without previous AMP injection (Second IVM; Figure 3E, right), since we hypothesized that our analysis method is sensitive enough to uncover a rotational bias even without AMP stressor. Indeed the effect observed after the first IVM injections could be re-induced with this second IVM injection at a lower dose and without AMP stimulation, reaching significance after 1 day (^*^p = 0.017, Mann Whitney test, GlyRα_1_^AG^ *n* = 5) and 2 days (^*^p = 0.017, Mann Whitney test), although a clear trend was already visible at 0.5 days post injection. The significant difference to control mice was lost after 4 days (*p* = 0.213, Mann Whitney test). Post-mortem analysis revealed that the expression of Venus was high in the injected brain region (Figure 3F left, see complete overview in (Supplementary Figure 4A), although targeting patterns varied between individual mice (Supplementary Figure 4C). Double immunolabeling and confocal imaging confirmed that cells, which expressed the fluorescent reporter, also contained GlyRα_1_^AG^ (Figure 3F, right). It also showed that the infection of injected regions was highly effective, since only a few cells (putative neurons) did not show detectable GlyRα_1_^AG^ or Venus fluorescence. Immunoblot analysis of tissue samples from ipsi- and contralateral striatal regions revealed a clear ipsilateral over-expression of GlyRα_1_^AG^ (Supplementary Figure 4B).

Together these results indicate that IVM had a reversible and re-inducible effect on mice expressing GlyRα_1_^AG^ in one of the striata. The rotational bias was highest between 0.5 and 1 days after IVM i.p. injection and returned to baseline in between 4 days (1.5 mg/kg IVM, −AMP) and 8 days (2.5 mg/kg IVM, +AMP).

### GlyRα_1_^AG^ mediated silencing of olfactory bulb neurons induces a slowing of gamma oscillations, a decrease in odor induced c-Fos expression, and impairments in odor discrimination

The consequences of GlyRα_1_^AG^ inhibition on neuronal network function were examined in the olfactory bulb (OB) of awake and freely moving mice. It has been shown that enhanced inhibition can lead to a decrease in oscillation frequency in the gamma range (40–100 Hz) in local OB networks (Bathellier et al., 2006). Comparable effects have also been observed in other brain regions after the injection of psychoactive drugs and upon direct pharmacological modulation of GABA_A_Rs (Whittington et al., 2000; Scheffzuk et al., 2013). By infecting OB neurons with our viral construct, we investigated whether OB fast oscillations can be modulated by the IVM induced inhibition of neurons expressing GlyRα_1_^AG^. We also wondered whether evoked alterations in OB network activity could be correlated with the performance in odor discrimination, which critically depends on the interplay of mitral/tufted cells and granule cells (Abraham et al., 2010).

**Figure 4:**
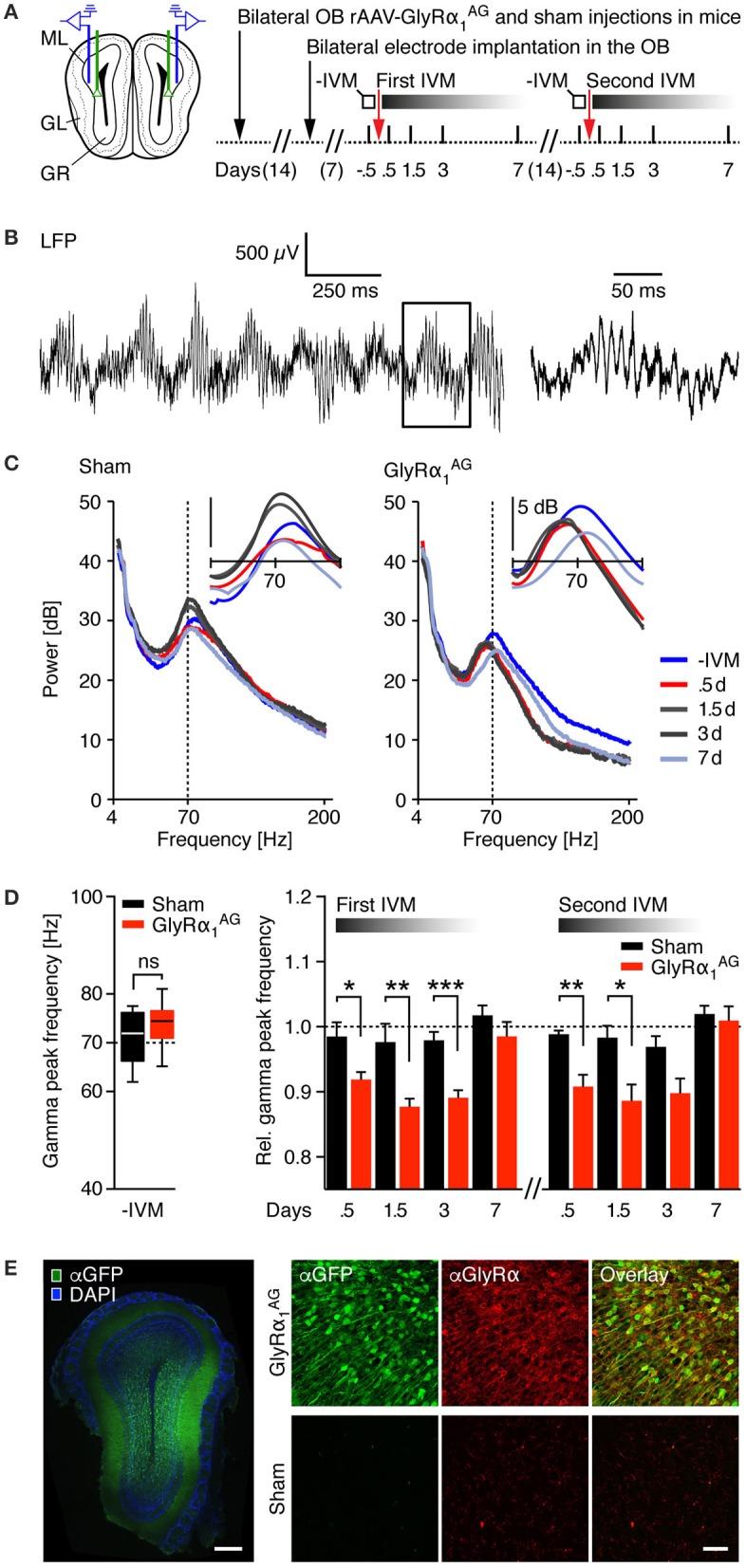
**IVM induced shifts toward slower gamma oscillations in the olfactory bulb of mice expressing GlyRα_**1**_AG. (A)** Left: Injection scheme; green lines indicate the path of the injection cannula for bilateral stereotactic rAAV-syn-GlyRα_1_^AG^-2A-Venus and sham (PBS) injections, and blue lines symbolize the position of the recording electrodes in the OB. Right: Time line of the experiment. Local field potentials (LFPs) were analyzed twice for 10 min in freely moving mice in an open arena on the days indicated. **(B)** Example raw signal of an intracranial LFP recording. Left: Fast oscillations overlying a slower rhythm are clearly visible. Right: Magnification of the box shown on the left shows fast oscillations in the gamma range. **(C)** Frequency analysis of LFPs (4–200 Hz) for one sham (left) and one GlyRα_1_^AG^ expressing mouse (right) over 10 min recordings; colors indicate recordings on different days over the time course of 1 week; vertical dashed line at 70 Hz is shown for reference. Inset: smoothed frequency analysis in the 40–100 Hz range. **(D)** Left: Baseline gamma peak frequencies (pooled first and second IVM injection); ns, not significant (*p* > 0.05); median, 25th/75th percentile. Right: Relative gamma peak frequencies normalized to baseline during 1 week after IVM; a substantial shift in the relative gamma peak frequency was observable 0.5, 1.5, and 3 days after IVM treatment in the OBs of rAAV-syn-GlyRα_1_^AG^-2A-Venus but not sham injected mice. First IVM: GlyRα_1_^AG^ *n* = 4, sham *n* = 4 (1.5d: GlyRα_1_^AG^ *n* = 3, sham *n* = 2); second IVM: GlyRα_1_^AG^ *n* = 3, sham *n* = 2. Data shown as mean ± SEM; ^*^p < 0.05; ^**^p < 0.01; ^***^p < 0.001. **(E)** Left: Stereotactic injection of rAAV-syn-GlyRα_1_^AG^-2A-Venus into the OB yielded broad expression of Venus (αGFP, green) at the injection site; counterstained for DAPI (blue); scale bar: 250 μm. Right: Confocal imaging revealed over-expression of GlyRα_1_^AG^ (αGlyRα, red) in Venus (αGFP, green) positive neurons; scale bar: 40 μm.

For the analysis of local field potentials (LFPs), stereotrodes were implanted on both sides of the OB in mice that had been bilaterally injected with either rAAV-syn-GlyRα_1_^AG^-2A-Venus or PBS (GlyRα_1_^AG^ *n* = 4, sham *n* = 4; Figure 4A, left). The implantations were targeted to the granule cell network, close to the mitral/tufted cell layer (Supplementary Figure 5A, left). After 1 week of recovery, stable recordings of intracranial LFPs were obtained over many days during the exploration of an open arena (Supplementary Figure 5A, right). Mice in both groups were injected with 2.5 mg/kg IVM 0.5 days prior to the first recording, and recurrent recordings were obtained over the following 7 days (Figure 4A, right). An example trace of the raw signal collected at one electrode can be seen in Figure 4B. As demonstrated in Figure 4C for one sham and one GlyRα_1_^AG^ expressing mouse, gamma peak frequencies stayed remarkably stable over all test days in the sham injected mouse, but shifted to lower frequencies 0.5–3 days after the injection of IVM in the GlyRα_1_^AG^ expressing mouse; all time points measured are shown in Figure 4D. Baseline gamma peak frequencies ranged from 62.1 to 77.6 Hz in sham and 65.3 to 81.1 Hz in GlyRα_1_^AG^ expressing mice (Figure 4D left, *p* = 0.33, Mann Whitney test). A decrease in gamma peak frequency was detected in LFP subsets sorted for either weak or strong responses to IVM in the rAAV-syn-GlyRα_1_^AG^-2A-Venus, but not the sham injected, mice (Figure 4D, right; Supplementary Figures 5C,D). Interestingly, the gamma peak frequency shifted back to its original values in IVM injected, GlyRα_1_^AG^ expressing mice within 7 days. Moreover, this gamma frequency shift could be re-induced with a second IVM injection and evolved over the same time course as observed after the first IVM exposure (Figure 4D, right; “Second IVM,” GlyRα_1_^AG^ *n* = 3, Sham *n* = 2; ^*^p < 0.05; ^**^p < 0.01, ^***^p < 0.001, Mann Whitney test). Notably, a comparable gamma peak frequency shift was observed in 3 control mice after the injection of diazepam (4 mg/kg), a positive allosteric modulator of GABA_A_Rs, which produced strong sedation (Supplementary Figure 5E). However, there were no differences in mobility seen after the injection of IVM between GlyRα_1_^AG^ and sham injected mice (Supplementary Figure 5B). Post-mortem analysis revealed a heterogeneous expression of Venus at the OB injection sites, spanning part of the granule cell network and the adjacent mitral/tufted cell layer (Figure 4E, left). This suggests that GlyRα_1_^AG^ mediated silencing was only effective in a subpopulation of neurons in the OB. GlyRα immunostaining confirmed the strong over-expression of GlyRα_1_^AG^ in Venus expressing cells (Figure 4E, right). Together these results disclose a specific, reversible and re-inducible effect of IVM on gamma oscillations in the OB of GlyRα_1_^AG^ expressing mice that was pronounced between 0.5 and 3 days after IVM delivery and reversed back to baseline within 7 days.

**Figure 5:**
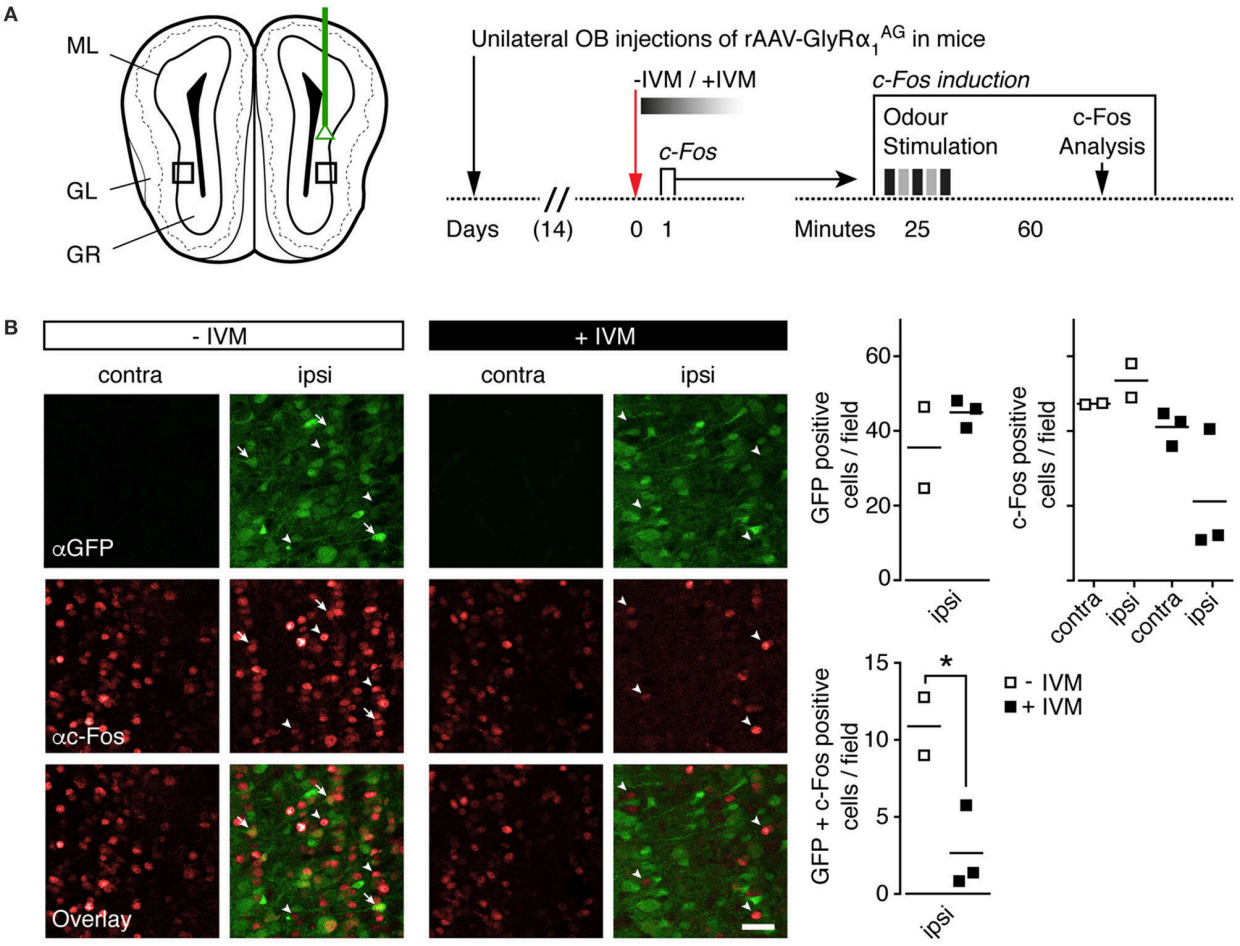
**IVM induced GlyRα_**1**_^**AG**^ activation lead to a decrease in odor induced c-Fos expression in the olfactory bulb**. **(A)** Left: Scheme of unilateral stereotactic OB injection of rAAV-syn-GlyRα_1_^AG^-2A-Venus. Black squares indicate approximate size and position of fields shown in **(B)**. ML, mitral/tufted cell layer; GL, glomerular layer; GR, granule cell layer. Right: Time line of the experiment; the c-Fos induction protocol used 1 day after IVM injection is shown on the right. **(B)** Left: Fields of 150 × 150 μm were evaluated for c-Fos (αc-Fos, red) and Venus (αGFP, green) expression. Without IVM injection (−IVM), the number of c-Fos immunoreactive cells was comparable on contra- and ipsilateral sides; after the injection of IVM (+IVM), the number of c-Fos immunoreactive cells and the number of GFP and c-Fos double labeled cells decreased steeply on the ipsilateral side (arrow tips: c-Fos only labeled cells, whole arrows: GFP/c-Fos double positive cells); scale bar: 30 μm. Right: Quantification of GFP and c-Fos positive cells (top row) and GFP/c-Fos double positive cells (bottom row), numbers per 150 × 150 μm; horizontal line indicates mean; GlyRα_1_^AG^ +IVM, *n* = 3; GlyRα_1_^AG^ −IVM, *n* = 2; ^*^p < 0.05.

In order to visualize the efficiency of IVM mediated inhibition in GlyRα_1_^AG^ expressing mice at the cellular level, we looked at odor induced c-Fos expression in the OB of a new cohort of mice with unilateral GlyRα_1_^AG^ expression (Figure 5A). Two weeks after recovery from virus injection, a single episode of strong odor exposure (see Section Materials and Methods) enhanced c-Fos expression in the ipsi- and contralateral bulbi of GlyRα_1_^AG^ expressing mice, which had received either PBS (“−IVM”) or 2.5 mg/kg IVM (“+IVM”) i.p. injections 1 day before the odor challenge (Supplementary Figure 6). However, closer inspection of the OB sections from IVM injected mice revealed weaker c-Fos immunostaining in those OB regions that exhibited strong expression of GlyRα_1_^AG^-2A-Venus (Supplementary Figure 6). Analysis of confocal images revealed a significantly lower number of GlyRα_1_^AG^-2A-Venus/c-Fos co-expressing cells after IVM as compared to PBS injection (Figure 5B, ^*^p = 0.0438, unpaired *t*-test, +IVM *n* = 3, −IVM *n* = 2). Thus, IVM induced silencing occurred specifically in OB neurons with high GlyRα_1_^AG^ levels and did not uniformly affect GlyRα_1_^AG^ expressing neuronal networks.

**Figure 6:**
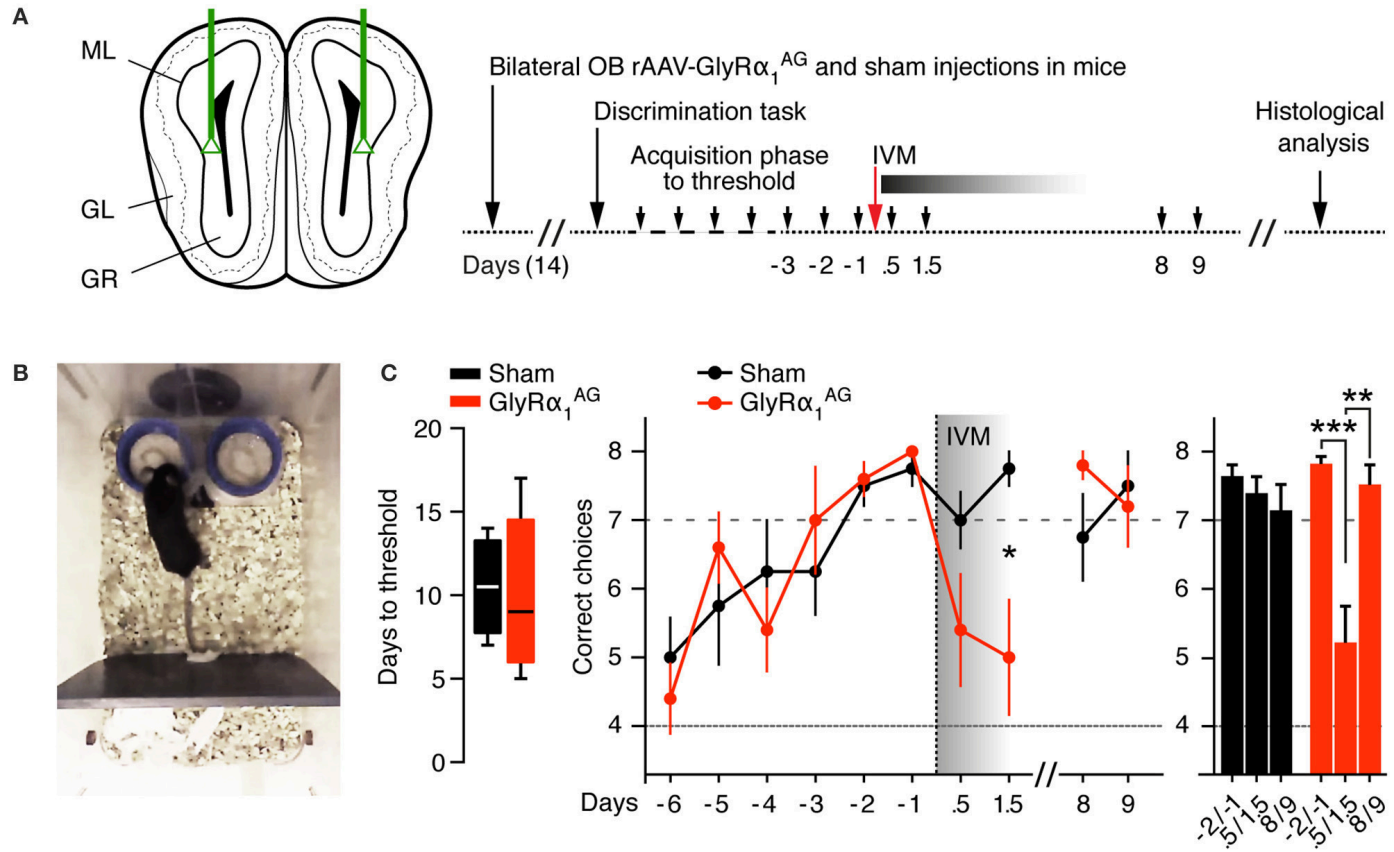
**IVM induced GlyRα_**1**_^**AG**^ activation in the olfactory bulb lead to a deficit in odor discrimination**. **(A)** Left: Scheme of bilateral stereotactic rAAV-syn-GlyRα_1_^AG^-2A-Venus and sham (PBS) injections into the OB. Right: Time line of the experiment. Small black arrows indicate days on which olfactory discrimination testing was performed. **(B)** Picture of the apparatus used in the discrimination test; the lids (blue) were scented with two odors of which only one was rewarded; both lids could be quickly removed from the apparatus if mice started to dig on the side with the wrong odor. **(C)** Left: The number of days in the acquisition phase required to reach the threshold criterion did not differ significantly in between groups (median, 25th/75th percentile, *p* > 0.05). Middle: 0.5–1.5 days after IVM injection, GlyRα_1_^AG^ expressing mice performed worse than on the 3 days prior to injection and compared to sham injected mice; mice recovered from this deficit 8 days after IVM injection. Right: Comparison of pairs of test days, each: -2 d and -1 d prior to, 0.5 d and 1.5 d after, and +8 d and +9 d after, IVM injection; GlyRα_1_^AG^ *n* = 5, Sham *n* = 4. Data shown as means ± SEM; ^*^p < 0.05, ^**^p < 0.01, ^***^p < 0.001.

To monitor odor recognition and discrimination, we trained another cohort of 5 mice 2 weeks after bilateral OB injection of rAAV-syn-GlyRα_1_^AG^-2A-Venus in an olfactory discrimination task (Mihalick et al., 2000) (Figures 6A,B; Supplementary Movie 2). All five mice reached the threshold criterion for correct odor discrimination (seven correct out of eight trials over 2 consecutive days) within time windows comparable to those seen with four sham (PBS) injected mice (*p* = 0.81, Mann Whitney test), indicating that virus injection and GlyRα_1_^AG^ expression in the OB did not interfere with odor recognition and discrimination (Figure 6C, left). However, when all mice were injected with 2.5 mg/kg IVM i.p. after they had reached the threshold, only GlyRα_1_^AG^ expressing animals showed a dramatic drop in odor discrimination performance 0.5–1.5 days later (Figure 6C, right, ^*^p = 0.024, Mann Whitney test). In line with our other results, this performance deficit was only transient and recovered fully after 7 days (*p* = 0.132, Mann Whitney test). Alterations in odor sensitivity and/or motivational aspects had no impact on the performance of mice in this discrimination task, since there were no significant differences found in decision times in between groups (Supplementary Figure 7A). Moreover, mice in both groups performed a pellet seeking task within comparable time spans before and 1.5 days after IVM injection (Supplementary Figure 7B). This suggests that the silencing of OB neurons specifically impaired performance within the odor discrimination task while leaving odor sensitivity unaffected.

## Discussion

In this study, we demonstrate that long-term reversible and re-inducible neuronal silencing via the IVM gated GlyRα_1_^AG^ (Lynagh and Lynch, 2010) can be used as an efficient tool for the investigation of neuronal networks and behavior. In acute hippocampal slices prepared from rAAV-syn-GlyRα_1_^AG^-2A-Venus injected mice, but not in slices from rAAV-syn-Venus injected mice, we could record an IVM induced hyperpolarization indicative of the functional expression of GlyRα_1_^AG^ in neurons of adult mice. This IVM induced hyperpolarization was accompanied by a strong decrease in AP frequency.

Intraperitoneal injection of 2.5 mg/kg IVM had no detectable effects on control mice but induced representative behavioral impairments in mice, which had received rAAV-syn-GlyRα_1_^AG^-2A-Venus injections into the striatum, hippocampus or olfactory bulb. In our experiments, the effects of GlyRα_1_^AG^ activation were detectable 12 h after IVM i.p. injection peaked between 24 and 36 h, and returned to baseline within 1 week; this is consistent with the time courses of IVM effects observed in virus-injected and transgenic animals (Lerchner et al., 2007; Hu et al., 2014). Although the IVM concentrations used in our study were lower than the ones employed in previous *in vivo* studies with IVM activated chloride channels (5 mg/kg) (Lerchner et al., 2007; Hu et al., 2014), we were able to generate effects of comparable size (Figure 3E, Second IVM: 1.5 mg/kg). This could be due to higher GlyRα_1_^AG^ expression levels achieved by delivery through rAAVs and synapsin promotor driven transcription. Importantly, the lower IVM dose used here should help to diminish possible IVM off-target effects on endogenous GABA_A_Rs and GlyRs (Adelsberger et al., 2000; Dawson et al., 2000; Shan et al., 2001). Despite the reduced IVM concentration, the behavioral effects of IVM induced GlyRα_1_^AG^ silencing reversed only after 4–8 days. Since the binding of IVM to GlyRs is irreversible (Shan et al., 2001), probably due to membrane integration of the lipophilic drug (Zemkova et al., 2014), the limiting factor for the recovery from silencing likely is a combination of rate-limited IVM clearance from the brain (Crichlow et al., 1986) and the replacement of GlyRα_1_^AG^ by newly translated receptors over time. We hypothesize that a further reduction of IVM concentrations might be possible which should accelerate the clearance of IVM liganded GlyRα_1_^AG^ and thereby shorten the overall time course of IVM dependent neuronal silencing. A shorter time window of GlyRα_1_^AG^ mediated inhibition should help to reduce the risk of homeostatic plasticity (Turrigiano, 1999) and permanent network changes that may occur when stimulatory inputs are removed (Margolis et al., 2012).

After recovery, neuronal silencing could be re-induced by a second IVM injection, as shown in the rotational assay (Striatum, Figure 3) and by recording gamma oscillations (OB, Figure 4), suggesting that GlyRα_1_^AG^ silenced neuronal networks do not undergo permanent changes. Importantly, we did not observe any obvious toxic effects of GlyRα_1_^AG^ expression on transduced neurons, and the activation of GlyRα_1_^AG^ with IVM did not lead to observable cell death or reactive gliosis.

We found that the induction of the immediate early gene c-Fos, a marker of neuronal activity (Clayton, 2000), was suppressed in GlyRα_1_^AG^ expressing olfactory bulb neurons after injection of IVM, and a quantification at the cellular scale revealed that this suppression was restricted to GlyRα_1_^AG^ transduced neurons and not a broader regional effect. This observation further underscores the specificity of our chemogenetic silencing approach. It also indicates that the silencing of a heterogeneous group of neurons was sufficient to promote a rotational phenotype, the observed SWM deficits and impairments in the odor discrimination task. Currently, we cannot discern whether the silencing of interneurons or excitatory neurons contributed to the observed phenotypes, since rAAV-syn-GlyRα_1_^AG^-2A-Venus is expressed in all classes of neurons. Nevertheless, the simultaneous silencing of principal and interneurons induced by IVM can lead to a transient imbalance in excitatory/inhibitory network drive, as disclosed by the shift in gamma oscillations seen in the GlyRα_1_^AG^ silenced OBs. Clearly, further experiments with cell-type specific expression of GlyRα_1_^AG^ are needed to dissect the specific network mechanism underlying these slowed gamma oscillations in the OB. We speculate that increased inhibition in a neuronal network as well as a lack thereof can lead to very similar impairments, as exemplified by the similar working memory impairments elicited through both optogenetic silencing of CA3 excitatory neurons (Shipton et al., 2014) and the lack of AMPAR subunits on parvalbumin-positive interneurons (Fuchs et al., 2007).

Neuronal silencing through the activation of virally transduced GlyRα_1_^AG^ as described here should be feasible in a wide range of experimental setups and model organisms because of the ease of delivery through rAAVs. Especially the ability to non-invasively modulate the activity of distributed neuronal networks in the brain over subsequent days makes GlyRα_1_^AG^ an attractive tool for behavioral research. While the precision of GlyRα_1_^AG^ mediated silencing in the temporal domain cannot be compared to current optogenetic methods, it can be considered superior in the spatial domain. Current optogenetic methods are not practical for the simultaneous control of extended brain regions and distributed neuronal ensembles, since sufficient illumination is required to activate them. This in turn requires the implantation of light conducting fibers, which increases the invasiveness of the experiment. As shown rather recently, transient manipulations as performed with optogenetic or fast pharmacological silencers might overestimate the steady-state function of a silenced neuronal network by evoking acute off-target effects in connected brain regions (Otchy et al., 2015). Thus, GlyRα_1_^AG^ mediated silencing, which reaches its peak effect on far slower time scales, might become an important tool for examining the function of a silenced neuronal network beyond acute disturbances in network homeostasis. Currently used chemogenetic silencers (hM4Di, KORD; Armbruster et al., 2007; Vardy et al., 2015) circumvent these problems in part, but act on faster time courses than GlyRα_1_^AG^ and therefore require frequent re-activation if used over the time course of hours and days (Ferguson et al., 2013; Vardy et al., 2015; Marchant et al., 2016). While these time courses might be desirable in some experimental setups, the stress of i.p. injection temporally close to behavioral testing is counterproductive for tasks involving decision making and other cognitive tests. The long onset and slow reversal of IVM mediated GlyRα_1_^AG^ silencing therefore represents an attractive alternative for silencing neuronal networks in multiple learning trials or over extended time periods.

## Author contributions

HB initiated, HO and RS designed, and HO executed and analyzed the experiments. AR performed patch clamp electrophysiology in acute hippocampal slices. IB performed the olfactory discrimination task. WT and HO generated the viral vectors. RS supervised the project. HO, AR, and RS wrote the manuscript. HB and JK commented and improved the manuscript.

## Funding

This study was supported by the Max Planck Society, grants from the German Research Foundation (SFB636/A4 and SFB1134/B01) to RS, and by the Program of Competitive Growth of Kazan Federal University and the subsidy allocated to Kazan Federal University for the state assignment in the sphere of scientific activities to AR.

### Conflict of interest statement

The authors declare that the research was conducted in the absence of any commercial or financial relationships that could be construed as a potential conflict of interest.
