# Supplementary material for: Causal Interrogation of Neuronal Networks and Behavior through Virally Transduced Ivermectin Receptors
Source: Front Mol Neurosci. 2016 Aug 30;9:75. doi: 10.3389/fnmol.2016.00075 (PMC5004486; doi:10.3389/fnmol.2016.00075)
Supplement: Supplementary file 3 [file SupplementaryFigures.pdf]

Supplementary Material

Causal interrogation of neuronal networks and behavior through virally transduced Ivermectin receptors

Horst Andreas Obenhaus, Andrei Rozov, Ilaria Bertocchi, Wannan Tang, Joachim Kirsch, Heinrich Betz, Rolf Sprengel*

***Correspondence:** Rolf Sprengel: Rolf.sprengel@mpimf-heidelberg.mpg.de

1 Supplementary Figures and Movies

1.1 Supplementary Figures

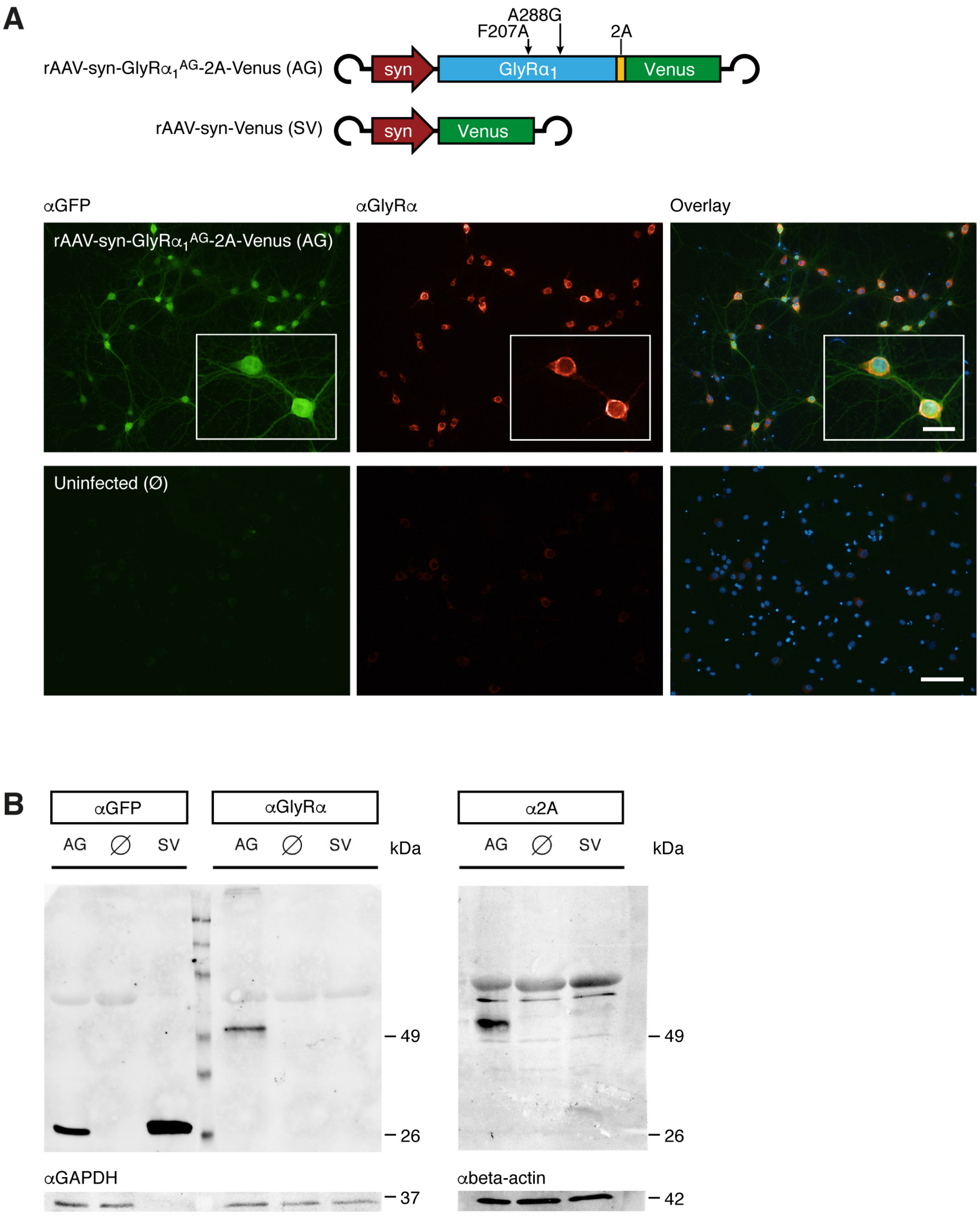

Supplementary Figure 1 | Immunofluorescence and immunoblot analysis of rAAV-syn-GlyRα_1^{AG}-2A-Venus infected rat hippocampal primary neurons. (**A**) Top: Schematic drawing of rAAVs. Bottom: Expression pattern of GlyRα_1^{AG} and Venus in rat hippocampal primary neurons infected with rAAV-syn-GlyRα_1^{AG}-2A-Venus (top row) and uninfected neurons as control (bottom row). From left to right: Venus (αGFP, green), GlyRα (αGlyRα, red), DAPI (blue); scale bar overview 100 µm, scale bar inset 25 µm. (**B**) Full size image of the immunoblot shown in Figure 1A for rAAV-syn-GlyRα_1^{AG}-2A-Venus (AG) infected, uninfected (∅) and rAAV-syn-Venus (SV) infected hippocampal neurons; αGlyRα: Glycine receptor alpha subunits, α2A: 2A peptide. Visualization of αGAPDH and αbeta-actin were used as loading controls. Please note that for αGFP

labelling the protein amount loaded for rAAV-syn-Venus infected cells loaded was only 1 μg as compared to 10 μg in all other lanes.

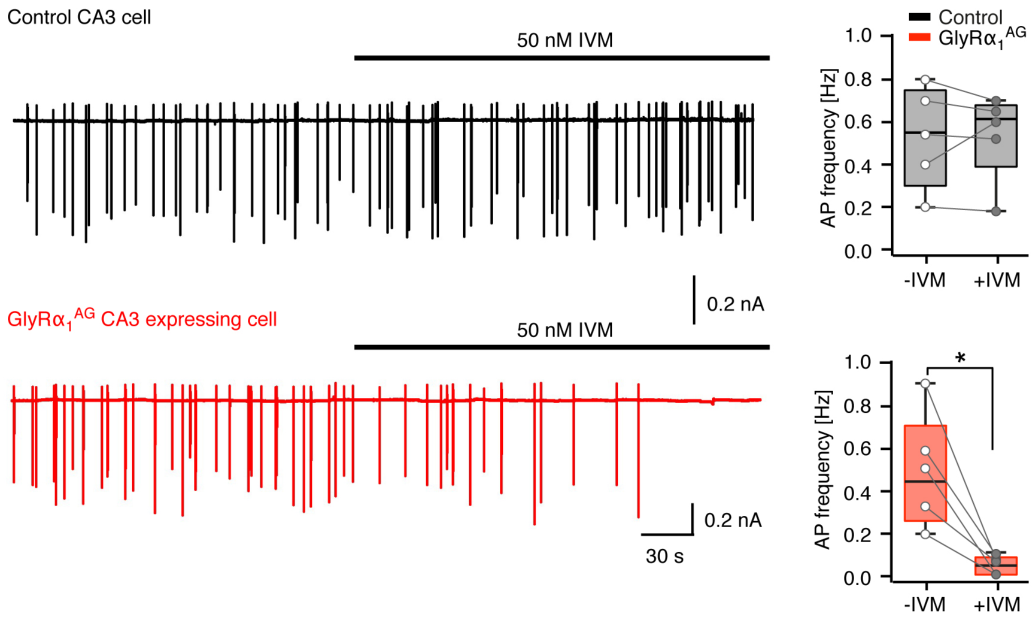

Supplementary Figure 2 | Cell attached recordings of CA3 hippocampal neurons in acute hippocampal slices. Effect of IVM administration on the action potential frequency of CA3 pyramidal cells detected by cell attached configuration. Left: Example traces of a control CA3 pyramidal cell (rAAV-syn-Venus injected mice, black trace) and a GlyRα_1^{AG} expressing CA3 pyramidal cell (rAAV-syn-GlyRα_1^{AG}-2A-Venus injected mice, red trace). Right: Box plots show cumulative data from control (n=5; top) and GlyRα_1^{AG} expressing (n=5; bottom) animals (median, $25^{th}/75^{th}$ percentile, $p^{*}<0.05$). The open and grey circles represent the AP frequency in individual experiments before and after IVM application, respectively.

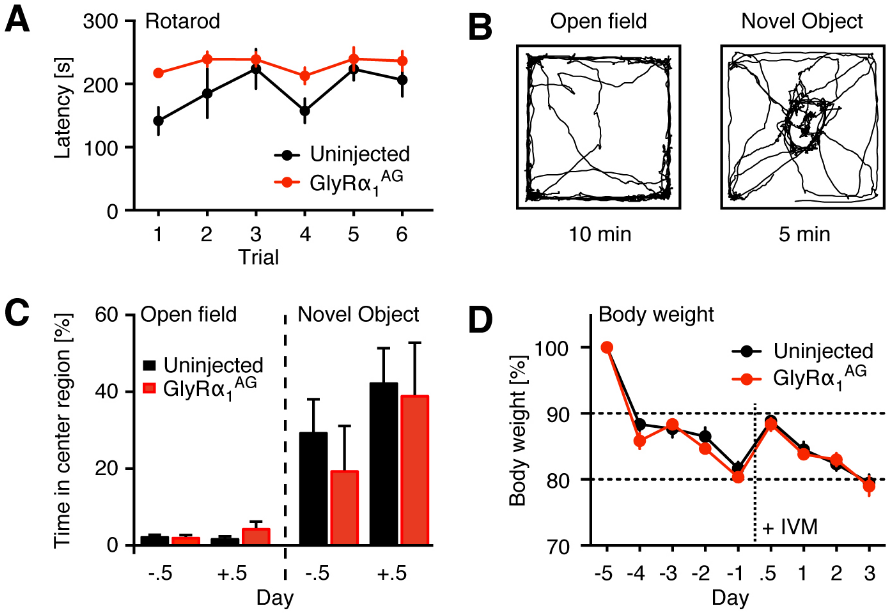

Supplementary Figure 3 | Behavioral performance of mice with bilateral hippocampal injection of rAAV-syn-GlyRα_1^{AG}-2A-Venus. (**A**) Rotarod performance did not differ between GlyRα_1^{AG} injected and virus uninjected control mice before IVM treatment. (**B**) Representative trace diagrams of the open field (10 min) and novel object exploration phase (5 min) of a virus uninjected mouse. (**C**) Quantification of the time spent in the center region showed that both GlyRα_1^{AG} injected and virus uninjected control mice similarly avoided the centre in the empty open field and explored the novel object in the novel object recognition test before (-0.5d) and after (+0.5d) IVM injection. (**D**) The body weight of all mice was closely monitored over the time course of the experiment (T-Maze task); all mice reached the fasting goal of <90% and >80% of their original body weight (%BW) in the days prior to testing (-5 to -1 days). GlyRα_1^{AG} n=6; uninjected n=6; all data shown as mean ± SEM.

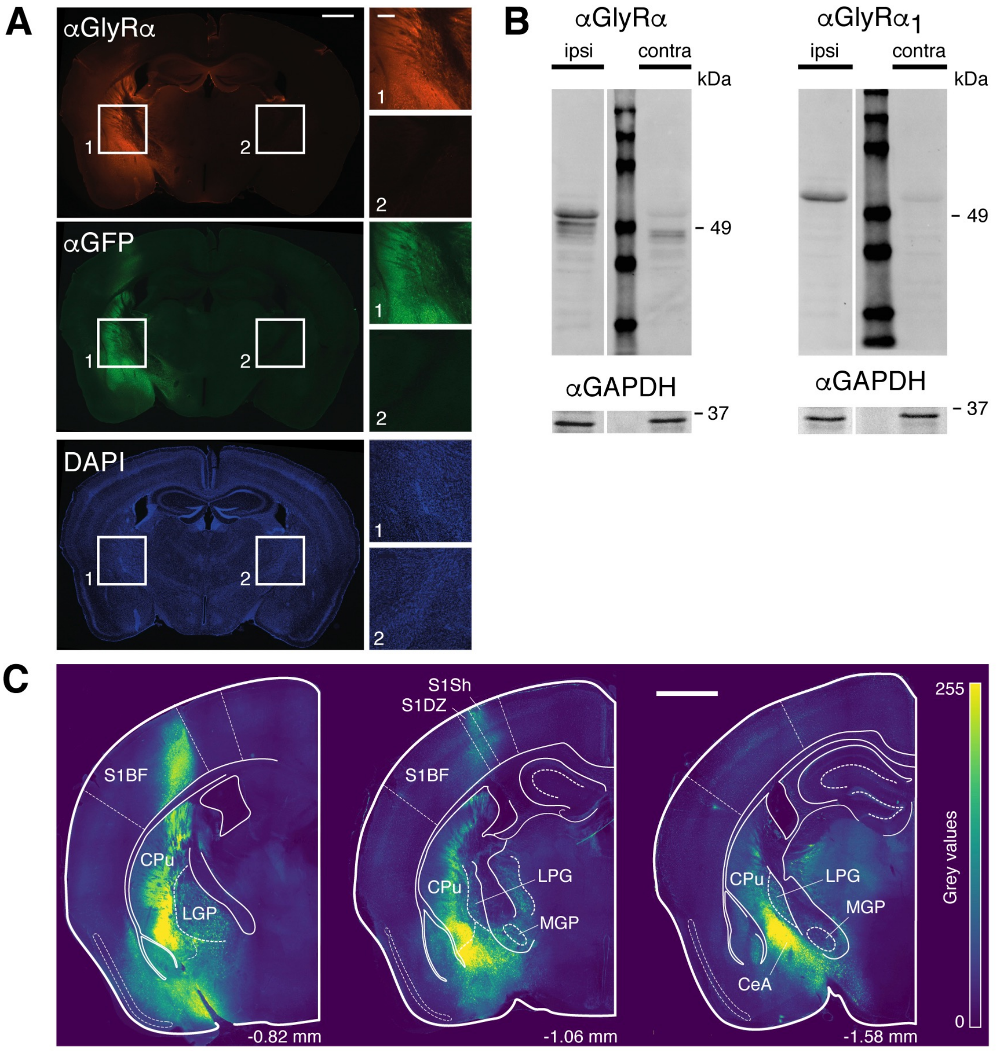

Supplementary Figure 4 | Unilateral striatal GlyRα$_1^{AG}$ expression. (**A**) Example coronal brain slice of one rAAV-syn-GlyRα$_1^{AG}$-2A-Venus injected mouse, stained for GlyRα$_1^{AG}$ (αGlyRα, red), Venus (αGFP, green) and DAPI (blue); scale bar overview 1 mm, scale bar inset: 250 µm. (**B**) Immunoblot of tissue homogenates of a rAAV-syn-GlyRα$_1^{AG}$-2A-Venus injected mouse showing over-expression of GlyRα$_1^{AG}$ in the injected (ipsi) versus the non-injected (contra) side of the brain; αGlyRα: glycine receptor alpha subunits, αGlyRα$_1$: glycine receptor alpha 1 subunits; 20 µg were loaded per lane. Multiple immunoreactive bands (< 50 kDa) were stained by αGlyRα, possibly due to the partial recognition of proteolysis products (Becker et al., 1993). (**C**) Pseudo-colored average intensity projections of Venus expression (αGFP); n=4 mice per average (one slice per mouse); distance from bregma is shown in millimeters; CPu: Caudate putamen; LGP: Lateral globus pallidus; MGP: Medial globus pallidus; CeA: Central amygdaloid nucleus; S1BF: Primary somatosensory cortex, barrel field; S1DZ: Primary somatosensory cortex, dysgranular region; S1Sh: Primary somatosensory cortex, shoulder region; scale bar: 1 mm.

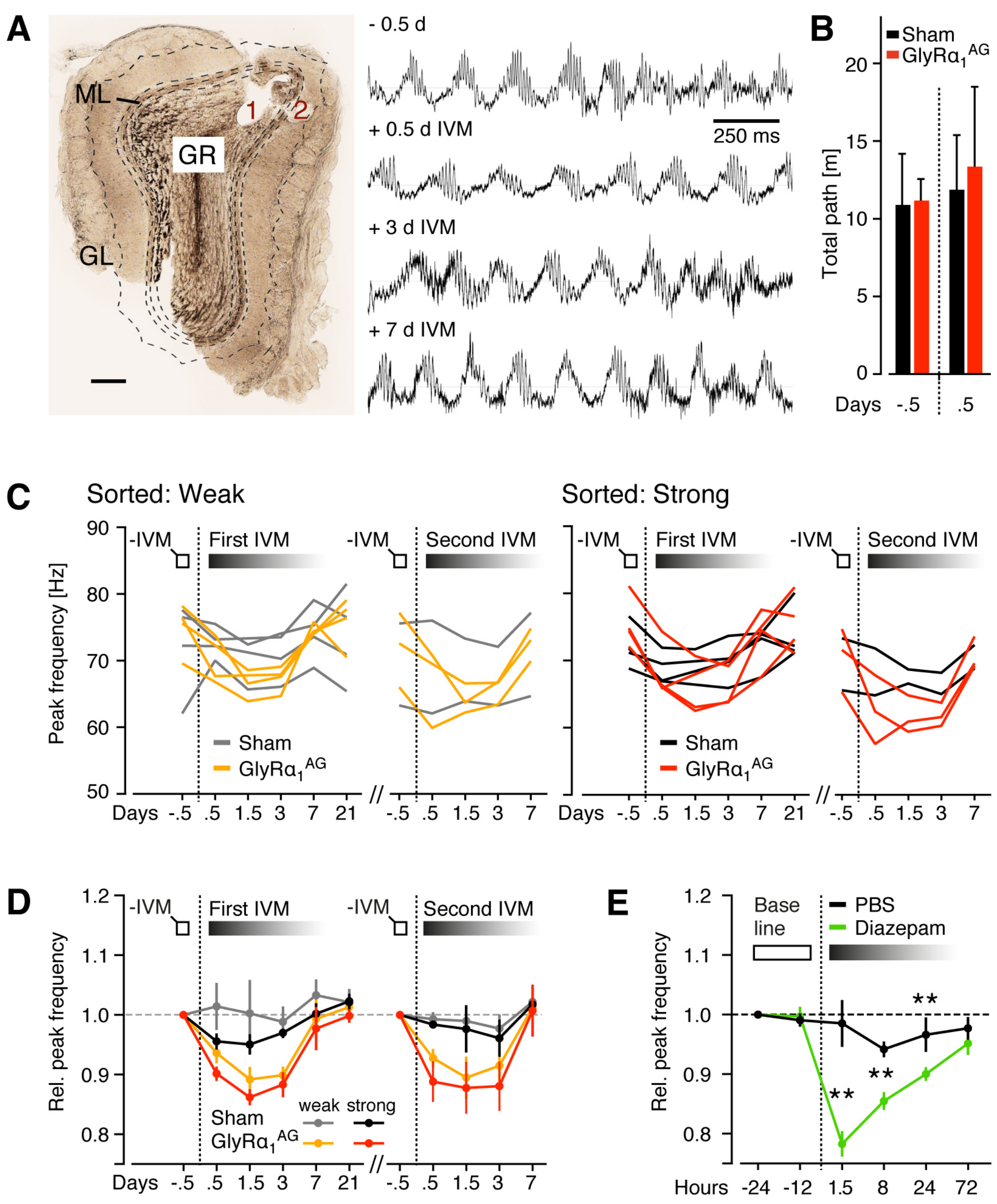

Supplementary Figure 5 | IVM induced shifts in the frequency of gamma oscillations in the OB of GlyRα1^{AG} expressing mice. (**A**) Left: Example brain slice showing the position of a stereotrode (1 and 2) after electrical lesion (*see methods*); electrode 1 lies within the granule cell layer (GR), electrode 2 touches the mitral/tufted cell layer (ML). GL: Glomerular layer; scale bar: 250 µm. Right: Raw LFP traces of a GlyRα1^{AG} expressing mouse; stable intracranial LFP signals were recorded over many days in the same mouse; phase/amplitude coupling of a slower rhythm (about 4 Hz, theta) and a faster rhythm (about 70 Hz, gamma) are readily visible on all days. (**B**) No significant differences in the path length travelled during the recording were found between groups, both before (-0.5 d) and after (+0.5 d) the injection of IVM (10 min, n=2 for sham and GlyRα1^{AG}); mean ± SEM. (**C**) Gamma peak frequencies extracted from recordings of the two halves of the olfactory bulb in every mouse were sorted (difference in absolute gamma peak frequency) according to their 'weak' and 'strong' reactions to IVM 0.5 days after the first and second i.p. IVM injections.

(**D**) The transient drop in gamma peak frequency seen over the first three days following IVM injection was detected in all LFPs recorded from GlyRα_1^{AG} expressing mice after the first and the second IVM injection (normalized to baseline, First IVM: GlyRα_1^{AG} n=4, Sham n=4 (1.5d: GlyRα_1^{AG} n=3, Sham n=2); Second IVM: GlyRα_1^{AG} n=3, Sham n=2); mean ± SEM. (**e**) The injection of Diazepam (4 mg/kg) lead to a similar shift in the gamma peak frequency as observed after IVM injection in GlyRα_1^{AG} expressing mice (normalized to baseline (-24 hours), Diazepam i.p. n=3, PBS i.p. n=2); mean ± SEM; p**<0.01.

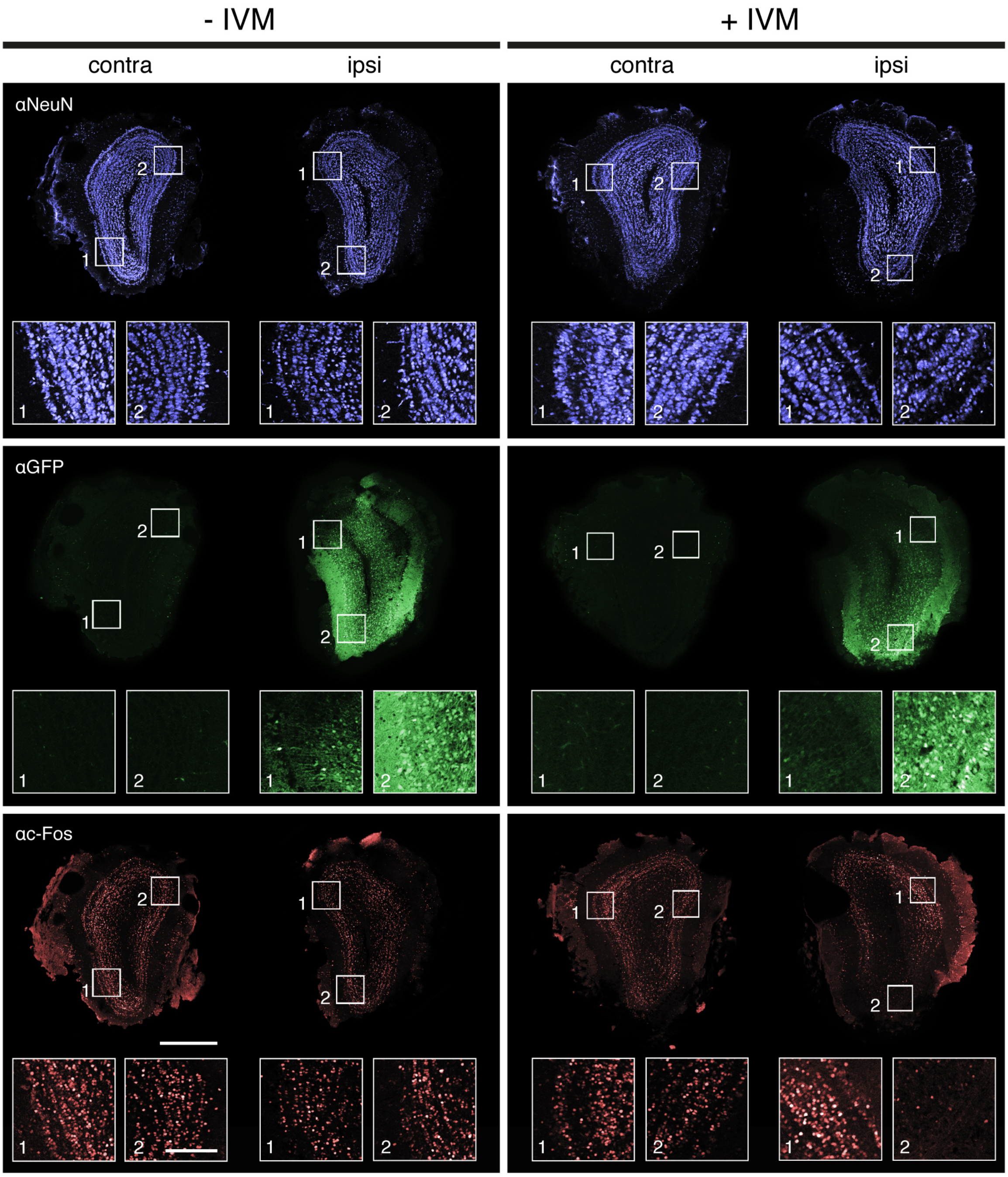

Supplementary Figure 6 | Odor induced c-Fos expression in the olfactory bulb of mice that were unilaterally injected with rAAV-syn-GlyRα_1^{AG}-2A-Venus decreases ipsilaterally after IVM injection. Confocal microscopy images of αNeuN (blue), αGFP (green) and αc-Fos (red) triple staining. The left two columns show OB slices of a mouse which had received PBS i.p. (-IVM), and the two right columns show OB slices of a mouse which had received IVM i.p. (+IVM) one day prior to c-Fos induction. A marked decrease in c-Fos expression was only visible after IVM injection in areas (insets) where rAAV-syn-GlyRα_1^{AG}-2A-Venus expression was high (see c-Fos signal in region no. 2, +IVM, ipsi). Scale bar overview: 500 µm, scale bar insets: 75 µm.

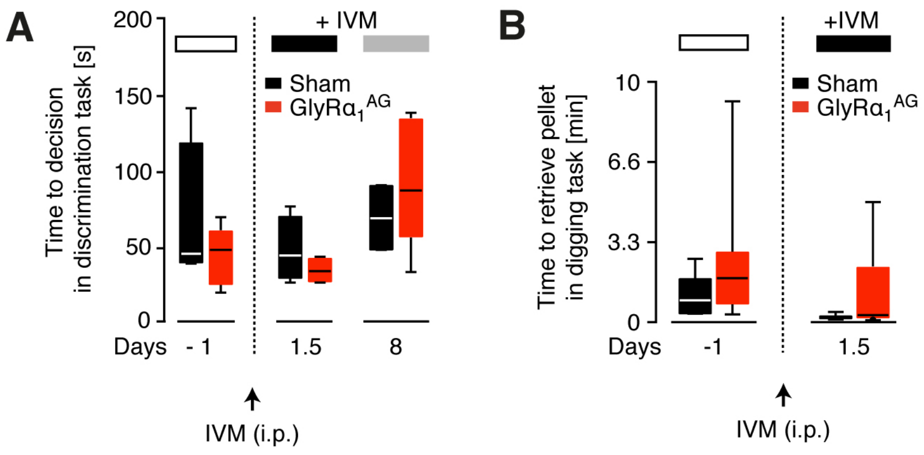

Supplementary Figure 7 | Behavioral deficits due to GlyRα1^{AG} mediated silencing of olfactory bulb networks spares reaction times in discrimination task and retrieval times in pellet seeking task. (**A**) Decision times in the odor discrimination task did not differ significantly in between groups before (-1 d), after (1.5 d) and one week after (8 d) IVM injection. (**B**) Retrieval times in the pellet seeking task did not differ significantly in between groups before (-1 d) or after (1.5 d) IVM injection; GlyRα1^{AG} n=5, sham n=4.

1.2 Supplementary Movies

Supplementary Movie 1 | Automatic extraction of rotational biases. Left: Original video; Right: Analysis overlay, showing the automatic extraction of three points on the mouse body (red dots) and the tail point (blue dot). This allowed the robust analysis of rotation angles for every analyzed video frame (boxed number shows extracted body angle (0-360°)).

Supplementary Movie 2 | Olfactory discrimination task. Two example trials of a mouse that has reached the threshold criterion. The mouse is confined to the home compartment (top) until the experimenter has finished preparing the sand-filled lids. It is then allowed to enter through the center and has to decide in which scented lid it starts to dig to retrieve the food reward. If the decision is correct (mouse starts digging in the lid with the correct odor) the trial ends rewarded, otherwise the rig with the two lids is retracted and no food reward is given (not shown). Video is sped up (about 6x real time).
